# Supplementary material for: OpenMM 7: Rapid development of high performance algorithms for molecular dynamics
Source: PLoS Comput Biol. 2017 Jul 26;13(7):e1005659. doi: 10.1371/journal.pcbi.1005659 (PMC5549999; doi:10.1371/journal.pcbi.1005659)
Supplement: S1 Source Code — (ZIP) [file pcbi.1005659.s002.zip › OpenMM-7.0.1-Source/examples/charmm22.rtf]

*>>>>>>>>CHARMM22 All-Hydrogen Topology File for Proteins <<<<<<

*>>>>> Includes phi, psi cross term map (CMAP) correction <<<<<<<

*>>>>>>>>>>>>>>>>>>>>>> December, 2003 <<<<<<<<<<<<<<<<<<<<<<<<<<

* All comments to ADM jr. via the CHARMM web site: www.charmm.org

*	parameter set discussion forum

*

31 1

! references

!

!PROTEINS

!

!MacKerell, A.D., Jr,. Feig, M., Brooks, C.L., III, Extending the

!treatment of backbone energetics in protein force fields: limitations

!of gas-phase quantum mechanics in reproducing protein conformational

!distributions in molecular dynamics simulations, Journal of

!Computational Chemistry, 25: 1400-1415, 2004.

!

!MacKerell, Jr., A. D.; Bashford, D.; Bellott, M.; Dunbrack Jr., R.L.;

!Evanseck, J.D.; Field, M.J.; Fischer, S.; Gao, J.; Guo, H.; Ha, S.;

!Joseph-McCarthy, D.; Kuchnir, L.; Kuczera, K.; Lau, F.T.K.; Mattos,

!C.; Michnick, S.; Ngo, T.; Nguyen, D.T.; Prodhom, B.; Reiher, III,

!W.E.; Roux, B.; Schlenkrich, M.; Smith, J.C.; Stote, R.; Straub, J.;

!Watanabe, M.; Wiorkiewicz-Kuczera, J.; Yin, D.; Karplus, M. All-atom

!empirical potential for molecular modeling and dynamics Studies of

!proteins. Journal of Physical Chemistry B, 1998, 102, 3586-3616.

!

!IONS (see lipid and nucleic acid topology and parameter files for

!additional ions

!

!ZINC

!

!Roland H. Stote and Martin Karplus, Zinc Binding in Proteins and

!Solution: A Simple but Accurate Nonbonded Representation, PROTEINS:

!Structure, Function, and Genetics 23:12-31 (1995)

!

MASS	1 H	1.00800 H ! polar H

MASS	2 HC	1.00800 H ! N-ter H

MASS	3 HA	1.00800 H ! nonpolar H

MASS	4 HT	1.00800 H ! TIPS3P WATER HYDROGEN

MASS	5 HP	1.00800 H ! aromatic H

MASS	6 HB	1.00800 H ! backbone H

MASS	7 HR1	1.00800 H ! his he1, (+) his HG,HD2

MASS	8 HR2	1.00800 H ! (+) his HE1

MASS	9 HR3	1.00800 H ! neutral his HG, HD2

MASS	10 HS	1.00800 H ! thiol hydrogen

MASS	11 HE1	1.00800 H ! for alkene; RHC=CR

MASS	12 HE2	1.00800 H ! for alkene; H2C=CR

MASS	13 HA1	1.00800 H ! alkane, CH, new LJ params (see toppar_all22_prot_aliphatic_c27.str)

MASS	14 HA2	1.00800 H ! alkane, CH2, new LJ params (see toppar_all22_prot_aliphatic_c27.str)

MASS	15 HA3	1.00800 H ! alkane, CH3, new LJ params (see toppar_all22_prot_aliphatic_c27.str)

MASS	16 HF1	1.00800 H ! Aliphatic H on fluorinated C (see toppar_all22_prot_fluoro_alkanes.str)

MASS	17 HF2	1.00800 H ! Aliphatic H on fluorinated C (see toppar_all22_prot_fluoro_alkanes.str)

MASS	20 C	12.01100 C ! carbonyl C, peptide backbone

MASS	21 CA	12.01100 C ! aromatic C

MASS	22 CT1	12.01100 C ! aliphatic sp3 C for CH

MASS	23 CT2	12.01100 C ! aliphatic sp3 C for CH2

MASS	24 CT3	12.01100 C ! aliphatic sp3 C for CH3

MASS	25 CPH1 12.01100 C ! his CG and CD2 carbons

MASS	26 CPH2 12.01100 C ! his CE1 carbon

MASS	27 CPT	12.01100 C ! trp C between rings

MASS	28 CY	12.01100 C ! TRP C in pyrrole ring

MASS	29 CP1	12.01100 C ! tetrahedral C (proline CA)

MASS	30 CP2	12.01100 C ! tetrahedral C (proline CB/CG)

MASS	31 CP3	12.01100 C ! tetrahedral C (proline CD)

MASS	32 CC	12.01100 C ! carbonyl C, asn,asp,gln,glu,cter,ct2

MASS	33 CD	12.01100 C ! carbonyl C, pres aspp,glup,ct1

MASS	34 CPA	12.01100 C ! heme alpha-C

MASS	35 CPB	12.01100 C ! heme beta-C

MASS	36 CPM	12.01100 C ! heme meso-C

MASS	37 CM	12.01100 C ! heme CO carbon

MASS	38 CS	12.01100 C ! thiolate carbon

MASS	39 CE1	12.01100 C ! for alkene; RHC=CR

MASS	40 CE2	12.01100 C ! for alkene; H2C=CR

MASS	41 CST	12.01100 C ! CO2 carbon

MASS	42 CT	12.01100 C ! aliphatic sp3 C, new LJ params, no hydrogens (see toppar_all22_prot_aliphatic_c27.str)

MASS	43 CT1x 12.01100 C ! aliphatic sp3 C for CH, new LJ params (see toppar_all22_prot_aliphatic_c27.str)

MASS	44 CT2x 12.01100 C ! aliphatic sp3 C for CH2, new LJ params (see toppar_all22_prot_aliphatic_c27.str)

MASS	45 CT3x 12.01100 C ! aliphatic sp3 C for CH3, new LJ params (see toppar_all22_prot_aliphatic_c27.str)

MASS	46 CN	12.01100 C ! C for cyano group (see toppar_all22_prot_pyridines.str)

MASS	47 CAP	12.01100 C ! aromatic C for pyrimidines (see toppar_all22_prot_pyridines.str)

MASS	48 COA	12.01100 C ! carbonyl C for pyrimidines (see toppar_all22_prot_pyridines.str)

MASS	49 C3	12.01100 C ! cyclopropyl carbon

MASS	50 N	14.00700 N ! proline N

MASS	51 NR1	14.00700 N ! neutral his protonated ring nitrogen

MASS	52 NR2	14.00700 N ! neutral his unprotonated ring nitrogen

MASS	53 NR3	14.00700 N ! charged his ring nitrogen

MASS	54 NH1	14.00700 N ! peptide nitrogen

MASS	55 NH2	14.00700 N ! amide nitrogen

MASS	56 NH3	14.00700 N ! ammonium nitrogen

MASS	57 NC2	14.00700 N ! guanidinium nitroogen

MASS	58 NY	14.00700 N ! TRP N in pyrrole ring

MASS	59 NP	14.00700 N ! Proline ring NH2+ (N-terminal)

MASS	60 NPH	14.00700 N ! heme pyrrole N

MASS	61 NC	14.00700 N ! N for cyano group (see toppar_all22_prot_pyridines.str)

MASS	70 O	15.99900 O ! carbonyl oxygen

MASS	71 OB	15.99900 O ! carbonyl oxygen in acetic acid

MASS	72 OC	15.99900 O ! carboxylate oxygen

MASS	73 OH1	15.99900 O ! hydroxyl oxygen

MASS	74 OS	15.99940 O ! ester oxygen

MASS	75 OT	15.99940 O ! TIPS3P WATER OXYGEN

MASS	76 OM	15.99900 O ! heme CO/O2 oxygen

MASS	77 OST	15.99900 O ! CO2 oxygen

MASS	78 OCA	15.99900 O ! carbonyl O for pyrimidines (see toppar_all22_prot_pyridines.str)

MASS	81 S	32.06000 S ! sulphur

MASS	82 SM	32.06000 S ! sulfur C-S-S-C type

MASS	83 SS	32.06000 S ! thiolate sulfur

MASS	85 HE	4.00260 HE ! helium

MASS	86 NE	20.17970 NE ! neon

MASS	87 CF1	12.01100 C ! monofluoromethyl (see toppar_all22_prot_fluoro_alkanes.str)

MASS	88 CF2	12.01100 C ! difluoromethyl (see toppar_all22_prot_fluoro_alkanes.str)

MASS	89 CF3	12.01100 C ! trifluoromethyl (see toppar_all22_prot_fluoro_alkanes.str)

MASS	90 FE	55.84700 Fe ! heme iron 56

MASS	91 CLAL 35.45300 CL ! Chlorine Atom (see toppar_all22_prot_aldehydes.str)

MASS	92 FA	18.99800 F ! aromatic flourine (see toppar_all22_prot_pyridines.str)

MASS	93 F1	18.99800 F ! Fluorine, monofluoro (see toppar_all22_prot_fluoro_alkanes.str)

MASS	94 F2	18.99800 F ! Fluorine, difluoro (see toppar_all22_prot_fluoro_alkanes.str)

MASS	95 F3	18.99800 F ! Fluorine, trifluoro (see toppar_all22_prot_fluoro_alkanes.str)

MASS	99 DUM	0.00000 H ! dummy atom

MASS	100 SOD 22.989770 NA ! Sodium Ion

MASS	101 MG	24.305000 MG ! Magnesium Ion

MASS	102 POT 39.102000 K ! Potassium Ion! check masses

MASS	103 CES 132.900000 CS ! Cesium Ion

MASS	104 CAL 40.080000 CA ! Calcium Ion

MASS	105 CLA 35.450000 CL ! Chloride Ion

MASS	106 ZN	65.370000 ZN ! zinc (II) cation

!the following masses must be added to the parent RTF for retinol/al

MASS	112 CC1A 12.01100 C ! alkene conjugation

MASS	113 CC1B 12.01100 C ! alkene conjugation

MASS	114 CC2	12.01100 C ! alkene conjugation

MASS	120 NS1	14.00700 N ! N for deprotonated Schiff's base

MASS	121 NS2	14.00700 N ! N for protonated Schiff's base

DECL -CA

DECL -C

DECL -O

DECL +N

DECL +HN

DECL +CA

DEFA FIRS NTER LAST CTER

AUTO ANGLES DIHE

RESI ALA	0.00

GROUP

ATOM N	NH1	-0.47 ! |

ATOM HN	H	0.31 ! HN-N

ATOM CA	CT1	0.07 ! |	HB1

ATOM HA	HB	0.09 ! |	/

GROUP	! HA-CA--CB-HB2

ATOM CB	CT3	-0.27 ! |	\

ATOM HB1 HA	0.09 ! |	HB3

ATOM HB2 HA	0.09 ! O=C

ATOM HB3 HA	0.09 ! |

GROUP	!

ATOM C	C	0.51

ATOM O	O	-0.51

BOND CB CA N HN N CA

BOND C CA C +N CA HA CB HB1 CB HB2 CB HB3

DOUBLE O C

IMPR N -C CA HN C CA +N O

CMAP -C N CA C	N CA C +N

DONOR HN N

ACCEPTOR O C

IC -C	CA	*N	HN	1.3551 126.4900 180.0000 115.4200 0.9996

IC -C	N	CA	C	1.3551 126.4900 180.0000 114.4400 1.5390

IC N	CA	C	+N	1.4592 114.4400 180.0000 116.8400 1.3558

IC +N	CA	*C	O	1.3558 116.8400 180.0000 122.5200 1.2297

IC CA	C	+N	+CA	1.5390 116.8400 180.0000 126.7700 1.4613

IC N	C	*CA CB	1.4592 114.4400 123.2300 111.0900 1.5461

IC N	C	*CA HA	1.4592 114.4400 -120.4500 106.3900 1.0840

IC C	CA	CB	HB1	1.5390 111.0900 177.2500 109.6000 1.1109

IC HB1 CA	*CB HB2	1.1109 109.6000 119.1300 111.0500 1.1119

IC HB1 CA	*CB HB3	1.1109 109.6000 -119.5800 111.6100 1.1114

RESI ARG	1.00

GROUP

ATOM N	NH1	-0.47 ! |	HH11

ATOM HN	H	0.31 ! HN-N	|

ATOM CA	CT1	0.07 ! |	HB1 HG1 HD1 HE	NH1-HH12

ATOM HA	HB	0.09 ! |	|	|	|	|	//(+)

GROUP	! HA-CA--CB--CG--CD--NE--CZ

ATOM CB	CT2	-0.18 ! |	|	|	|	\

ATOM HB1 HA	0.09 ! |	HB2 HG2 HD2	NH2-HH22

ATOM HB2 HA	0.09 ! O=C	|

GROUP	! |	HH21

ATOM CG	CT2	-0.18

ATOM HG1 HA	0.09

ATOM HG2 HA	0.09

GROUP

ATOM CD	CT2	0.20

ATOM HD1 HA	0.09

ATOM HD2 HA	0.09

ATOM NE	NC2	-0.70

ATOM HE	HC	0.44

ATOM CZ	C	0.64

ATOM NH1 NC2	-0.80

ATOM HH11 HC	0.46

ATOM HH12 HC	0.46

ATOM NH2 NC2	-0.80

ATOM HH21 HC	0.46

ATOM HH22 HC	0.46

GROUP

ATOM C	C	0.51

ATOM O	O	-0.51

BOND CB CA CG CB CD CG NE CD CZ NE

BOND NH2 CZ N HN N CA

BOND C	CA C +N CA HA CB HB1

BOND CB HB2 CG HG1 CG HG2 CD HD1 CD HD2

BOND NE HE NH1 HH11 NH1 HH12 NH2 HH21 NH2 HH22

DOUBLE O C	CZ NH1

IMPR N -C CA HN	C CA +N O

CMAP -C N CA C	N CA C +N

IMPR CZ NH1 NH2 NE

DONOR HN N

DONOR HE NE

DONOR HH11 NH1

DONOR HH12 NH1

DONOR HH21 NH2

DONOR HH22 NH2

ACCEPTOR O C

IC -C	CA	*N	HN	1.3496 122.4500 180.0000 116.6700 0.9973

IC -C	N	CA	C	1.3496 122.4500 180.0000 109.8600 1.5227

IC N	CA	C	+N	1.4544 109.8600 180.0000 117.1200 1.3511

IC +N	CA	*C	O	1.3511 117.1200 180.0000 121.4000 1.2271

IC CA	C	+N	+CA	1.5227 117.1200 180.0000 124.6700 1.4565

IC N	C	*CA CB	1.4544 109.8600 123.6400 112.2600 1.5552

IC N	C	*CA HA	1.4544 109.8600 -117.9300 106.6100 1.0836

IC N	CA	CB	CG	1.4544 110.7000 180.0000 115.9500 1.5475

IC CG	CA	*CB HB1	1.5475 115.9500 120.0500 106.4000 1.1163

IC CG	CA	*CB HB2	1.5475 115.9500 -125.8100 109.5500 1.1124

IC CA	CB	CG	CD	1.5552 115.9500 180.0000 114.0100 1.5384

IC CD	CB	*CG HG1	1.5384 114.0100 125.2000 108.5500 1.1121

IC CD	CB	*CG HG2	1.5384 114.0100 -120.3000 108.9600 1.1143

IC CB	CG	CD	NE	1.5475 114.0100 180.0000 107.0900 1.5034

IC NE	CG	*CD HD1	1.5034 107.0900 120.6900 109.4100 1.1143

IC NE	CG	*CD HD2	1.5034 107.0900 -119.0400 111.5200 1.1150

IC CG	CD	NE	CZ	1.5384 107.0900 180.0000 123.0500 1.3401

IC CZ	CD	*NE HE	1.3401 123.0500 180.0000 113.1400 1.0065

IC CD	NE	CZ	NH1	1.5034 123.0500 180.0000 118.0600 1.3311

IC NE	CZ	NH1 HH11 1.3401 118.0600 -178.2800 120.6100 0.9903

IC HH11 CZ	*NH1 HH12 0.9903 120.6100 171.1900 116.2900 1.0023

IC NH1 NE	*CZ NH2	1.3311 118.0600 178.6400 122.1400 1.3292

IC NE	CZ	NH2 HH21 1.3401 122.1400 -174.1400 119.9100 0.9899

IC HH21 CZ	*NH2 HH22 0.9899 119.9100 166.1600 116.8800 0.9914

RESI ASN	0.00

GROUP

ATOM N	NH1	-0.47 ! |

ATOM HN	H	0.31 ! HN-N

ATOM CA	CT1	0.07 ! |	HB1 OD1	HD21 (cis to OD1)

ATOM HA	HB	0.09 ! |	|	||	/

GROUP	! HA-CA--CB--CG--ND2

ATOM CB	CT2	-0.18 ! |	|	\

ATOM HB1 HA	0.09 ! |	HB2	HD22 (trans to OD1)

ATOM HB2 HA	0.09 ! O=C

GROUP	! |

ATOM CG	CC	0.55

ATOM OD1 O	-0.55

GROUP

ATOM ND2 NH2	-0.62

ATOM HD21 H	0.32

ATOM HD22 H	0.30

GROUP

ATOM C	C	0.51

ATOM O	O	-0.51

BOND CB CA CG CB	ND2 CG

BOND N HN N CA	C	CA	C +N

BOND CA HA CB HB1 CB HB2 ND2 HD21 ND2 HD22

DOUBLE C O	CG OD1

IMPR N	-C CA	HN	C	CA +N	O

IMPR CG ND2 CB	OD1	CG CB ND2 OD1

IMPR ND2 CG HD21 HD22 ND2 CG HD22 HD21

CMAP -C N CA C	N CA C +N

DONOR HN N

DONOR HD21 ND2

DONOR HD22 ND2

ACCEPTOR OD1 CG

ACCEPTOR O C

IC -C	CA	*N	HN	1.3480 124.0500 180.0000 114.4900 0.9992

IC -C	N	CA	C	1.3480 124.0500 180.0000 105.2300 1.5245

IC N	CA	C	+N	1.4510 105.2300 180.0000 117.3800 1.3467

IC +N	CA	*C	O	1.3467 117.3800 180.0000 120.3200 1.2282

IC CA	C	+N	+CA	1.5245 117.3800 180.0000 124.8800 1.4528

IC N	C	*CA CB	1.4510 105.2300 121.1800 113.0400 1.5627

IC N	C	*CA HA	1.4510 105.2300 -115.5200 107.6300 1.0848

IC N	CA	CB	CG	1.4510 110.9100 180.0000 114.3000 1.5319

IC CG	CA	*CB HB1	1.5319 114.3000 119.1700 107.8200 1.1120

IC CG	CA	*CB HB2	1.5319 114.3000 -123.7400 110.3400 1.1091

IC CA	CB	CG	OD1	1.5627 114.3000 180.0000 122.5600 1.2323

IC OD1 CB	*CG ND2	1.2323 122.5600 -179.1900 116.1500 1.3521

IC CB	CG	ND2 HD21 1.5319 116.1500 -179.2600 117.3500 0.9963

IC HD21 CG	*ND2 HD22 0.9963 117.3500 178.0200 120.0500 0.9951

RESI ASP	-1.00

GROUP

ATOM N	NH1	-0.47 ! |

ATOM HN	H	0.31 ! HN-N

ATOM CA	CT1	0.07 ! |	HB1	OD1

ATOM HA	HB	0.09 ! |	|	//

GROUP	! HA-CA--CB--CG

ATOM CB	CT2	-0.28 ! |	|	\

ATOM HB1 HA	0.09 ! |	HB2	OD2(-)

ATOM HB2 HA	0.09 ! O=C

ATOM CG	CC	0.62 ! |

ATOM OD1 OC	-0.76

ATOM OD2 OC	-0.76

GROUP

ATOM C	C	0.51

ATOM O	O	-0.51

BOND CB CA CG CB OD2 CG

BOND N HN N CA	C	CA C +N

BOND CA HA CB HB1 CB HB2

DOUBLE O	C	CG OD1

IMPR N	-C CA HN C CA +N O

!IMPR OD1 CB OD2 CG

IMPR CG CB OD2 OD1

CMAP -C N CA C	N CA C +N

DONOR HN N

ACCEPTOR OD1 CG

ACCEPTOR OD2 CG

ACCEPTOR O C

IC -C	CA	*N	HN	1.3465 125.3100 180.0000 112.9400 0.9966

IC -C	N	CA	C	1.3465 125.3100 180.0000 105.6300 1.5315

IC N	CA	C	+N	1.4490 105.6300 180.0000 117.0600 1.3478

IC +N	CA	*C	O	1.3478 117.0600 180.0000 120.7100 1.2330

IC CA	C	+N	+CA	1.5315 117.0600 180.0000 125.3900 1.4484

IC N	C	*CA CB	1.4490 105.6300 122.3300 114.1000 1.5619

IC N	C	*CA HA	1.4490 105.6300 -116.4000 106.7700 1.0841

IC N	CA	CB	CG	1.4490 111.1000 180.0000 112.6000 1.5218

IC CG	CA	*CB HB1	1.5218 112.6000 119.2200 109.2300 1.1086

IC CG	CA	*CB HB2	1.5218 112.6000 -121.6100 110.6400 1.1080

IC CA	CB	CG	OD1	1.5619 112.6000 180.0000 117.9900 1.2565

IC OD1 CB	*CG OD2	1.2565 117.9900 -170.2300 117.7000 1.2541

RESI CYS	0.00

GROUP

ATOM N	NH1	-0.47 ! |

ATOM HN	H	0.31 ! HN-N

ATOM CA	CT1	0.07 ! |	HB1

ATOM HA	HB	0.09 ! |	|

GROUP	! HA-CA--CB--SG

ATOM CB	CT2	-0.11 ! |	|	\

ATOM HB1 HA	0.09 ! |	HB2	HG1

ATOM HB2 HA	0.09 ! O=C

ATOM SG	S	-0.23 ! |

ATOM HG1 HS	0.16

GROUP

ATOM C	C	0.51

ATOM O	O	-0.51

BOND CB CA	SG CB	N HN N CA

BOND C CA	C +N CA HA CB HB1

BOND CB HB2 SG HG1

DOUBLE O C

IMPR N -C CA HN C CA +N O

CMAP -C N CA C	N CA C +N

DONOR HN N

DONOR HG1 SG

ACCEPTOR O C

IC -C	CA	*N	HN	1.3479 123.9300 180.0000 114.7700 0.9982

IC -C	N	CA	C	1.3479 123.9300 180.0000 105.8900 1.5202

IC N	CA	C	+N	1.4533 105.8900 180.0000 118.3000 1.3498

IC +N	CA	*C	O	1.3498 118.3000 180.0000 120.5900 1.2306

IC CA	C	+N	+CA	1.5202 118.3000 180.0000 124.5000 1.4548

IC N	C	*CA CB	1.4533 105.8900 121.7900 111.9800 1.5584

IC N	C	*CA HA	1.4533 105.8900 -116.3400 107.7100 1.0837

IC N	CA	CB	SG	1.4533 111.5600 180.0000 113.8700 1.8359

IC SG	CA	*CB HB1	1.8359 113.8700 119.9100 107.2400 1.1134

IC SG	CA	*CB HB2	1.8359 113.8700 -125.3200 109.8200 1.1124

IC CA	CB	SG	HG1	1.5584 113.8700 176.9600 97.1500 1.3341

RESI GLN	0.00

GROUP

ATOM N	NH1	-0.47 ! |

ATOM HN	H	0.31 ! HN-N

ATOM CA	CT1	0.07 ! |	HB1 HG1 OE1	HE21 (cis to OE1)

ATOM HA	HB	0.09 ! |	|	|	||	/

GROUP	! HA-CA--CB--CG--CD--NE2

ATOM CB	CT2	-0.18 ! |	|	|	\

ATOM HB1 HA	0.09 ! |	HB2 HG2	HE22 (trans to OE1)

ATOM HB2 HA	0.09 ! O=C

GROUP	! |

ATOM CG	CT2	-0.18

ATOM HG1 HA	0.09

ATOM HG2 HA	0.09

GROUP

ATOM CD	CC	0.55

ATOM OE1 O	-0.55

GROUP

ATOM NE2 NH2	-0.62

ATOM HE21 H	0.32

ATOM HE22 H	0.30

GROUP

ATOM C	C	0.51

ATOM O	O	-0.51

BOND CB CA CG CB	CD CG	NE2 CD

BOND N HN N	CA	C	CA

BOND C +N CA HA	CB HB1 CB HB2 CG HG1

BOND CG HG2 NE2 HE21 NE2 HE22

DOUBLE O C	CD OE1

IMPR N	-C CA	HN	C	CA +N	O

IMPR CD NE2 CG	OE1	CD CG NE2 OE1

IMPR NE2 CD HE21 HE22 NE2 CD HE22 HE21

CMAP -C N CA C	N CA C +N

DONOR HN N

DONOR HE21 NE2

DONOR HE22 NE2

ACCEPTOR OE1 CD

ACCEPTOR O C

IC -C	CA	*N	HN	1.3477 123.9300 180.0000 114.4500 0.9984

IC -C	N	CA	C	1.3477 123.9300 180.0000 106.5700 1.5180

IC N	CA	C	+N	1.4506 106.5700 180.0000 117.7200 1.3463

IC +N	CA	*C	O	1.3463 117.7200 180.0000 120.5900 1.2291

IC CA	C	+N	+CA	1.5180 117.7200 180.0000 124.3500 1.4461

IC N	C	*CA CB	1.4506 106.5700 121.9100 111.6800 1.5538

IC N	C	*CA HA	1.4506 106.5700 -116.8200 107.5300 1.0832

IC N	CA	CB	CG	1.4506 111.4400 180.0000 115.5200 1.5534

IC CG	CA	*CB HB1	1.5534 115.5200 120.9300 106.8000 1.1147

IC CG	CA	*CB HB2	1.5534 115.5200 -124.5800 109.3400 1.1140

IC CA	CB	CG	CD	1.5538 115.5200 180.0000 112.5000 1.5320

IC CD	CB	*CG HG1	1.5320 112.5000 118.6900 110.4100 1.1112

IC CD	CB	*CG HG2	1.5320 112.5000 -121.9100 110.7400 1.1094

IC CB	CG	CD	OE1	1.5534 112.5000 180.0000 121.5200 1.2294

IC OE1 CG	*CD NE2	1.2294 121.5200 179.5700 116.8400 1.3530

IC CG	CD	NE2 HE21 1.5320 116.8400 -179.7200 116.8600 0.9959

IC HE21 CD	*NE2 HE22 0.9959 116.8600 -178.9100 119.8300 0.9943

RESI GLU	-1.00

GROUP

ATOM N	NH1	-0.47 ! |

ATOM HN	H	0.31 ! HN-N

ATOM CA	CT1	0.07 ! |	HB1 HG1	OE1

ATOM HA	HB	0.09 ! |	|	|	//

GROUP	! HA-CA--CB--CG--CD

ATOM CB	CT2	-0.18 ! |	|	|	\

ATOM HB1 HA	0.09 ! |	HB2 HG2	OE2(-)

ATOM HB2 HA	0.09 ! O=C

GROUP	! |

ATOM CG	CT2	-0.28

ATOM HG1 HA	0.09

ATOM HG2 HA	0.09

ATOM CD	CC	0.62

ATOM OE1 OC	-0.76

ATOM OE2 OC	-0.76

GROUP

ATOM C	C	0.51

ATOM O	O	-0.51

BOND CB CA CG CB CD CG OE2 CD

BOND N HN N CA C	CA

BOND C +N CA HA CB HB1 CB HB2 CG HG1

BOND CG HG2

DOUBLE O C	CD OE1

IMPR N	-C CA HN C CA +N O

IMPR CD CG OE2 OE1

CMAP -C N CA C	N CA C +N

DONOR HN N

ACCEPTOR OE1 CD

ACCEPTOR OE2 CD

ACCEPTOR O C

IC -C	CA	*N	HN	1.3471 124.4500 180.0000 113.9900 0.9961

IC -C	N	CA	C	1.3471 124.4500 180.0000 107.2700 1.5216

IC N	CA	C	+N	1.4512 107.2700 180.0000 117.2500 1.3501

IC +N	CA	*C	O	1.3501 117.2500 180.0000 121.0700 1.2306

IC CA	C	+N	+CA	1.5216 117.2500 180.0000 124.3000 1.4530

IC N	C	*CA CB	1.4512 107.2700 121.9000 111.7100 1.5516

IC N	C	*CA HA	1.4512 107.2700 -118.0600 107.2600 1.0828

IC N	CA	CB	CG	1.4512 111.0400 180.0000 115.6900 1.5557

IC CG	CA	*CB HB1	1.5557 115.6900 121.2200 108.1600 1.1145

IC CG	CA	*CB HB2	1.5557 115.6900 -123.6500 109.8100 1.1131

IC CA	CB	CG	CD	1.5516 115.6900 180.0000 115.7300 1.5307

IC CD	CB	*CG HG1	1.5307 115.7300 117.3800 109.5000 1.1053

IC CD	CB	*CG HG2	1.5307 115.7300 -121.9600 111.0000 1.1081

IC CB	CG	CD	OE1	1.5557 115.7300 180.0000 114.9900 1.2590

IC OE1 CG	*CD OE2	1.2590 114.9900 -179.1000 120.0800 1.2532

RESI GLY	0.00

GROUP

ATOM N	NH1	-0.47 ! |

ATOM HN	H	0.31 ! N-H

ATOM CA	CT2	-0.02 ! |

ATOM HA1 HB	0.09 ! |

ATOM HA2 HB	0.09 ! HA1-CA-HA2

GROUP	! |

ATOM C	C	0.51 ! |

ATOM O	O	-0.51 ! C=O

! |

BOND N HN N CA C CA

BOND C +N CA HA1 CA HA2

DOUBLE O C

IMPR N -C CA HN C CA	+N O

CMAP -C N CA C	N CA C +N

DONOR HN N

ACCEPTOR O C

IC -C	CA	*N	HN	1.3475 122.8200 180.0000 115.6200 0.9992

IC -C	N	CA	C	1.3475 122.8200 180.0000 108.9400 1.4971

IC N	CA	C	+N	1.4553 108.9400 180.0000 117.6000 1.3479

IC +N	CA	*C	O	1.3479 117.6000 180.0000 120.8500 1.2289

IC CA	C	+N	+CA	1.4971 117.6000 180.0000 124.0800 1.4560

IC N	C	*CA HA1	1.4553 108.9400 117.8600 108.0300 1.0814

IC N	C	*CA HA2	1.4553 108.9400 -118.1200 107.9500 1.0817

PATCHING FIRS GLYP

RESI HSD	0.00 ! neutral HIS, proton on ND1

GROUP

ATOM N	NH1	-0.47 ! |	HD1	HE1

ATOM HN	H	0.31 ! HN-N	|	/

ATOM CA	CT1	0.07 ! |	HB1	ND1--CE1

ATOM HA	HB	0.09 ! |	|	/	||

GROUP	! HA-CA--CB--CG	||

ATOM CB	CT2	-0.09 ! |	|	\\	||

ATOM HB1 HA	0.09 ! |	HB2	CD2--NE2

ATOM HB2 HA	0.09 ! O=C	|

ATOM ND1 NR1	-0.36 ! |	HD2

ATOM HD1 H	0.32

ATOM CG	CPH1	-0.05

GROUP

ATOM CE1 CPH2	0.25

ATOM HE1 HR1	0.13

ATOM NE2 NR2	-0.70

ATOM CD2 CPH1	0.22

ATOM HD2 HR3	0.10

GROUP

ATOM C	C	0.51

ATOM O	O	-0.51

BOND CB CA	CG CB	ND1 CG	CE1 ND1

BOND NE2 CD2 N	HN	N	CA

BOND C	CA	C	+N	CA HA	CB HB1

BOND CB HB2 ND1 HD1 CD2 HD2 CE1 HE1

DOUBLE O C	CG CD2	CE1 NE2

IMPR ND1 CG CE1 HD1 CD2 CG NE2 HD2 CE1 ND1 NE2 HE1

IMPR ND1 CE1 CG HD1 CD2 NE2 CG HD2 CE1 NE2 ND1 HE1

IMPR N	-C CA HN	C	CA +N O

CMAP -C N CA C	N CA C +N

DONOR HN N

DONOR HD1 ND1

ACCEPTOR NE2

ACCEPTOR O C

IC -C	CA	*N	HN	1.3475 123.2700 180.0000 115.2100 0.9988

IC -C	N	CA	C	1.3475 123.2700 180.0000 107.7000 1.5166

IC N	CA	C	+N	1.4521 107.7000 180.0000 117.5700 1.3509

IC +N	CA	*C	O	1.3509 117.5700 180.0000 120.2400 1.2273

IC CA	C	+N	+CA	1.5166 117.5700 180.0000 123.7200 1.4545

IC N	C	*CA CB	1.4521 107.7000 122.4600 109.9900 1.5519

IC N	C	*CA HA	1.4521 107.7000 -117.4900 107.3700 1.0830

IC N	CA	CB	CG	1.4521 112.1200 180.0000 114.0500 1.5041

IC CG	CA	*CB HB1	1.5041 114.0500 121.1700 109.0100 1.1118

IC CG	CA	*CB HB2	1.5041 114.0500 -122.3600 109.5300 1.1121

IC CA	CB	CG	ND1	1.5519 114.0500	90.0000 124.1000 1.3783

IC ND1 CB	*CG CD2	1.3783 124.1000 -171.2900 129.6000 1.3597

IC CB	CG	ND1 CE1	1.5041 124.1000 -173.2100 107.0300 1.3549

IC CB	CG	CD2 NE2	1.5041 129.6000 171.9900 110.0300 1.3817

IC NE2 ND1 *CE1 HE1	1.3166 111.6300 -179.6300 123.8900 1.0932

IC CE1 CG	*ND1 HD1	1.3549 107.0300 -174.6500 126.2600 1.0005

IC NE2 CG	*CD2 HD2	1.3817 110.0300 -177.8500 129.6300 1.0834

RESI HSE	0.00 ! neutral His, proton on NE2

GROUP

ATOM N	NH1	-0.47 ! |	HE1

ATOM HN	H	0.31 ! HN-N	__ /

ATOM CA	CT1	0.07 ! |	HB1	ND1--CE1

ATOM HA	HB	0.09 ! |	|	/	|

GROUP	! HA-CA--CB--CG	|

ATOM CB	CT2	-0.08 ! |	|	\\	|

ATOM HB1 HA	0.09 ! |	HB2	CD2--NE2

ATOM HB2 HA	0.09 ! O=C	|	\

ATOM ND1 NR2	-0.70 ! |	HD2	HE2

ATOM CG	CPH1	0.22

ATOM CE1 CPH2	0.25

ATOM HE1 HR1	0.13

GROUP

ATOM NE2 NR1	-0.36

ATOM HE2 H	0.32

ATOM CD2 CPH1	-0.05

ATOM HD2 HR3	0.09

GROUP

ATOM C	C	0.51

ATOM O	O	-0.51

BOND CB CA	CG CB	ND1 CG

BOND NE2 CD2 N	HN	N	CA

BOND C	CA	C	+N	NE2 CE1 CA HA	CB HB1

BOND CB HB2 NE2 HE2 CD2 HD2 CE1 HE1

DOUBLE	O	C	CD2 CG	CE1 ND1

IMPR NE2 CD2 CE1 HE2 CD2 CG NE2 HD2 CE1 ND1 NE2 HE1

IMPR NE2 CE1 CD2 HE2 CD2 NE2 CG HD2 CE1 NE2 ND1 HE1

IMPR N	-C CA HN	C	CA +N O

CMAP -C N CA C	N CA C +N

DONOR HN N

DONOR HE2 NE2

ACCEPTOR ND1

ACCEPTOR O C

IC -C	CA	*N	HN	1.3472 124.1600 180.0000 114.3600 0.9991

IC -C	N	CA	C	1.3472 124.1600 180.0000 106.4300 1.5166

IC N	CA	C	+N	1.4532 106.4300 180.0000 116.9700 1.3446

IC +N	CA	*C	O	1.3446 116.9700 180.0000 120.6800 1.2290

IC CA	C	+N	+CA	1.5166 116.9700 180.0000 124.9500 1.4505

IC N	C	*CA CB	1.4532 106.4300 123.5200 111.6700 1.5578

IC N	C	*CA HA	1.4532 106.4300 -116.4900 107.0800 1.0833

IC N	CA	CB	CG	1.4532 112.8200 180.0000 116.9400 1.5109

IC CG	CA	*CB HB1	1.5109 116.9400 119.8000 107.9100 1.1114

IC CG	CA	*CB HB2	1.5109 116.9400 -124.0400 109.5000 1.1101

IC CA	CB	CG	ND1	1.5578 116.9400	90.0000 120.1700 1.3859

IC ND1 CB	*CG CD2	1.3859 120.1700 -178.2600 129.7100 1.3596

IC CB	CG	ND1 CE1	1.5109 120.1700 -179.2000 105.2000 1.3170

IC CB	CG	CD2 NE2	1.5109 129.7100 178.6600 105.8000 1.3782

IC NE2 ND1 *CE1 HE1	1.3539 111.7600 179.6900 124.5800 1.0929

IC CE1 CD2 *NE2 HE2	1.3539 107.1500 -178.6900 125.8600 0.9996

IC NE2 CG	*CD2 HD2	1.3782 105.8000 -179.3500 129.8900 1.0809

RESI HSP	1.00 ! Protonated His

GROUP

ATOM N	NH1	-0.47 ! |	HD1	HE1

ATOM HN	H	0.31 ! HN-N	|	/

ATOM CA	CT1	0.07 ! |	HB1	ND1--CE1

ATOM HA	HB	0.09 ! |	|	/	||

GROUP	! HA-CA--CB--CG	||

ATOM CB	CT2	-0.05 ! |	|	\\	||

ATOM HB1 HA	0.09 ! |	HB2	CD2--NE2(+)

ATOM HB2 HA	0.09 ! O=C	|	\

ATOM CD2 CPH1	0.19 ! |	HD2	HE2

ATOM HD2 HR1	0.13

ATOM CG	CPH1	0.19

GROUP

ATOM NE2 NR3	-0.51

ATOM HE2 H	0.44

ATOM ND1 NR3	-0.51

ATOM HD1 H	0.44

ATOM CE1 CPH2	0.32

ATOM HE1 HR2	0.18

GROUP

ATOM C	C	0.51

ATOM O	O	-0.51

BOND CB CA	CG CB	ND1 CG	CE1 ND1

BOND NE2 CD2 N	HN	N	CA

BOND C	CA	C	+N CA HA CB HB1

BOND CB HB2 ND1 HD1 NE2 HE2 CD2 HD2 CE1 HE1

DOUBLE O	C	CD2 CG	NE2 CE1

IMPR ND1 CG CE1 HD1 ND1 CE1 CG HD1

IMPR NE2 CD2 CE1 HE2 NE2 CE1 CD2 HE2

IMPR N	-C CA HN	C	CA +N O

CMAP -C N CA C	N CA C +N

DONOR HN N

DONOR HD1 ND1

DONOR HE2 NE2

ACCEPTOR O C

IC -C	CA	*N	HN	1.3489 123.9300 180.0000 118.8000 1.0041

IC -C	N	CA	C	1.3489 123.9300 180.0000 112.0300 1.5225

IC N	CA	C	+N	1.4548 112.0300 180.0000 116.4900 1.3464

IC +N	CA	*C	O	1.3464 116.4900 180.0000 121.2000 1.2284

IC CA	C	+N	+CA	1.5225 116.4900 180.0000 124.2400 1.4521

IC N	C	*CA CB	1.4548 112.0300 125.1300 109.3800 1.5533

IC N	C	*CA HA	1.4548 112.0300 -119.2000 106.7200 1.0832

IC N	CA	CB	CG	1.4548 112.2500 180.0000 114.1800 1.5168

IC CG	CA	*CB HB1	1.5168 114.1800 122.5000 108.9900 1.1116

IC CG	CA	*CB HB2	1.5168 114.1800 -121.5100 108.9700 1.1132

IC CA	CB	CG	ND1	1.5533 114.1800	90.0000 122.9400 1.3718

IC ND1 CB	*CG CD2	1.3718 122.9400 -165.2600 128.9300 1.3549

IC CB	CG	ND1 CE1	1.5168 122.9400 -167.6200 108.9000 1.3262

IC CB	CG	CD2 NE2	1.5168 128.9300 167.1300 106.9300 1.3727

IC NE2 ND1 *CE1 HE1	1.3256 108.5000 178.3900 125.7600 1.0799

IC CE1 CD2 *NE2 HE2	1.3256 108.8200 -172.9400 125.5200 1.0020

IC CE1 CG	*ND1 HD1	1.3262 108.9000 171.4900 126.0900 1.0018

IC NE2 CG	*CD2 HD2	1.3727 106.9300 -174.4900 128.4100 1.0867

RESI ILE	0.00

GROUP

ATOM N	NH1	-0.47 ! |	HG21 HG22

ATOM HN	H	0.31 ! HN-N	| /

ATOM CA	CT1	0.07 ! |	CG2--HG23

ATOM HA	HB	0.09 ! |	/

GROUP	! HA-CA--CB-HB	HD1

ATOM CB	CT1	-0.09 ! |	\	/

ATOM HB	HA	0.09 ! |	CG1--CD--HD2

GROUP	! O=C	/ \	\

ATOM CG2 CT3	-0.27 ! | HG11 HG12 HD3

ATOM HG21 HA	0.09

ATOM HG22 HA	0.09

ATOM HG23 HA	0.09

GROUP

ATOM CG1 CT2	-0.18

ATOM HG11 HA	0.09

ATOM HG12 HA	0.09

GROUP

ATOM CD	CT3	-0.27

ATOM HD1 HA	0.09

ATOM HD2 HA	0.09

ATOM HD3 HA	0.09

GROUP

ATOM C	C	0.51

ATOM O	O	-0.51

BOND CB CA	CG1 CB	CG2 CB	CD CG1

BOND N	HN	N	CA	C	CA	C	+N

BOND CA HA	CB HB	CG1 HG11 CG1 HG12 CG2 HG21

BOND CG2 HG22 CG2 HG23 CD HD1 CD HD2 CD HD3

DOUBLE O	C

IMPR N -C CA HN C CA +N O

CMAP -C N CA C	N CA C +N

DONOR HN N

ACCEPTOR O C

IC -C	CA	*N	HN	1.3470 124.1600 180.0000 114.1900 0.9978

IC -C	N	CA	C	1.3470 124.1600 180.0000 106.3500 1.5190

IC N	CA	C	+N	1.4542 106.3500 180.0000 117.9700 1.3465

IC +N	CA	*C	O	1.3465 117.9700 180.0000 120.5900 1.2300

IC CA	C	+N	+CA	1.5190 117.9700 180.0000 124.2100 1.4467

IC N	C	*CA CB	1.4542 106.3500 124.2200 112.9300 1.5681

IC N	C	*CA HA	1.4542 106.3500 -115.6300 106.8100 1.0826

IC N	CA	CB	CG1	1.4542 112.7900 180.0000 113.6300 1.5498

IC CG1 CA	*CB HB	1.5498 113.6300 114.5500 104.4800 1.1195

IC CG1 CA	*CB CG2	1.5498 113.6300 -130.0400 113.9300 1.5452

IC CA	CB	CG2 HG21 1.5681 113.9300 -171.3000 110.6100 1.1100

IC HG21 CB	*CG2 HG22 1.1100 110.6100 119.3500 110.9000 1.1102

IC HG21 CB	*CG2 HG23 1.1100 110.6100 -120.0900 110.9700 1.1105

IC CA	CB	CG1 CD	1.5681 113.6300 180.0000 114.0900 1.5381

IC CD	CB	*CG1 HG11 1.5381 114.0900 122.3600 109.7800 1.1130

IC CD	CB	*CG1 HG12 1.5381 114.0900 -120.5900 108.8900 1.1141

IC CB	CG1 CD	HD1	1.5498 114.0900 -176.7800 110.3100 1.1115

IC HD1 CG1 *CD HD2	1.1115 110.3100 119.7500 110.6500 1.1113

IC HD1 CG1 *CD HD3	1.1115 110.3100 -119.7000 111.0200 1.1103

RESI LEU	0.00

GROUP

ATOM N	NH1	-0.47 ! |	HD11 HD12

ATOM HN	H	0.31 ! HN-N	| /

ATOM CA	CT1	0.07 ! |	HB1	CD1--HD13

ATOM HA	HB	0.09 ! |	|	/

GROUP	! HA-CA--CB--CG-HG

ATOM CB	CT2	-0.18 ! |	|	\

ATOM HB1 HA	0.09 ! |	HB2	CD2--HD23

ATOM HB2 HA	0.09 ! O=C	| \

GROUP	! |	HD21 HD22

ATOM CG	CT1	-0.09

ATOM HG	HA	0.09

GROUP

ATOM CD1 CT3	-0.27

ATOM HD11 HA	0.09

ATOM HD12 HA	0.09

ATOM HD13 HA	0.09

GROUP

ATOM CD2 CT3	-0.27

ATOM HD21 HA	0.09

ATOM HD22 HA	0.09

ATOM HD23 HA	0.09

GROUP

ATOM C	C	0.51

ATOM O	O	-0.51

BOND CB CA	CG CB	CD1 CG	CD2 CG

BOND N	HN	N	CA	C	CA	C +N

BOND CA HA	CB HB1 CB HB2 CG HG	CD1 HD11

BOND CD1 HD12 CD1 HD13 CD2 HD21 CD2 HD22 CD2 HD23

DOUBLE	O	C

IMPR N -C CA HN C CA +N O

CMAP -C N CA C	N CA C +N

DONOR HN N

ACCEPTOR O C

IC -C	CA	*N	HN	1.3474 124.3100 180.0000 114.2600 0.9979

IC -C	N	CA	C	1.3474 124.3100 180.0000 106.0500 1.5184

IC N	CA	C	+N	1.4508 106.0500 180.0000 117.9300 1.3463

IC +N	CA	*C	O	1.3463 117.9300 180.0000 120.5600 1.2299

IC CA	C	+N	+CA	1.5184 117.9300 180.0000 124.2600 1.4467

IC N	C	*CA CB	1.4508 106.0500 121.5200 112.1200 1.5543

IC N	C	*CA HA	1.4508 106.0500 -116.5000 107.5700 1.0824

IC N	CA	CB	CG	1.4508 111.1900 180.0000 117.4600 1.5472

IC CG	CA	*CB HB1	1.5472 117.4600 120.9800 107.1700 1.1145

IC CG	CA	*CB HB2	1.5472 117.4600 -124.6700 108.9800 1.1126

IC CA	CB	CG	CD1	1.5543 117.4600 180.0000 110.4800 1.5361

IC CD1 CB	*CG CD2	1.5361 110.4800 120.0000 112.5700 1.5360

IC CD1 CD2 *CG HG	1.5361 110.2600 120.0000 108.0200 1.1168

IC CB	CG	CD1 HD11 1.5472 110.4800 177.3300 110.5400 1.1111

IC HD11 CG	*CD1 HD12 1.1111 110.5400 119.9600 110.6200 1.1112

IC HD11 CG	*CD1 HD13 1.1111 110.5400 -119.8500 110.6900 1.1108

IC CB	CG	CD2 HD21 1.5472 112.5700 178.9600 110.3200 1.1116

IC HD21 CG	*CD2 HD22 1.1116 110.3200 119.7100 111.6900 1.1086

IC HD21 CG	*CD2 HD23 1.1116 110.3200 -119.6100 110.4900 1.1115

RESI LYS	1.00

GROUP

ATOM N	NH1	-0.47 ! |

ATOM HN	H	0.31 ! HN-N

ATOM CA	CT1	0.07 ! |	HB1 HG1 HD1 HE1	HZ1

ATOM HA	HB	0.09 ! |	|	|	|	|	/

GROUP	! HA-CA--CB--CG--CD--CE--NZ--HZ2

ATOM CB	CT2	-0.18 ! |	|	|	|	|	\

ATOM HB1 HA	0.09 ! |	HB2 HG2 HD2 HE2	HZ3

ATOM HB2 HA	0.09 ! O=C

GROUP	! |

ATOM CG	CT2	-0.18

ATOM HG1 HA	0.09

ATOM HG2 HA	0.09

GROUP

ATOM CD	CT2	-0.18

ATOM HD1 HA	0.09

ATOM HD2 HA	0.09

GROUP

ATOM CE	CT2	0.21

ATOM HE1 HA	0.05

ATOM HE2 HA	0.05

ATOM NZ	NH3	-0.30

ATOM HZ1 HC	0.33

ATOM HZ2 HC	0.33

ATOM HZ3 HC	0.33

GROUP

ATOM C	C	0.51

ATOM O	O	-0.51

BOND CB CA	CG CB	CD CG	CE CD	NZ CE

BOND N HN	N CA	C CA

BOND C +N	CA HA	CB HB1 CB HB2 CG HG1

BOND CG HG2 CD HD1 CD HD2 CE HE1 CE HE2

DOUBLE	O C

BOND NZ HZ1 NZ HZ2 NZ HZ3

IMPR N -C CA HN C CA +N O

CMAP -C N CA C	N CA C +N

DONOR HN N

DONOR HZ1 NZ

DONOR HZ2 NZ

DONOR HZ3 NZ

ACCEPTOR O C

IC -C	CA	*N	HN	1.3482 123.5700 180.0000 115.1100 0.9988

IC -C	N	CA	C	1.3482 123.5700 180.0000 107.2900 1.5187

IC N	CA	C	+N	1.4504 107.2900 180.0000 117.2700 1.3478

IC +N	CA	*C	O	1.3478 117.2700 180.0000 120.7900 1.2277

IC CA	C	+N	+CA	1.5187 117.2700 180.0000 124.9100 1.4487

IC N	C	*CA CB	1.4504 107.2900 122.2300 111.3600 1.5568

IC N	C	*CA HA	1.4504 107.2900 -116.8800 107.3600 1.0833

IC N	CA	CB	CG	1.4504 111.4700 180.0000 115.7600 1.5435

IC CG	CA	*CB HB1	1.5435 115.7600 120.9000 107.1100 1.1146

IC CG	CA	*CB HB2	1.5435 115.7600 -124.4800 108.9900 1.1131

IC CA	CB	CG	CD	1.5568 115.7600 180.0000 113.2800 1.5397

IC CD	CB	*CG HG1	1.5397 113.2800 120.7400 109.1000 1.1138

IC CD	CB	*CG HG2	1.5397 113.2800 -122.3400 108.9900 1.1143

IC CB	CG	CD	CE	1.5435 113.2800 180.0000 112.3300 1.5350

IC CE	CG	*CD HD1	1.5350 112.3300 122.2500 108.4100 1.1141

IC CE	CG	*CD HD2	1.5350 112.3300 -121.5900 108.1300 1.1146

IC CG	CD	CE	NZ	1.5397 112.3300 180.0000 110.4600 1.4604

IC NZ	CD	*CE HE1	1.4604 110.4600 119.9100 110.5100 1.1128

IC NZ	CD	*CE HE2	1.4604 110.4600 -120.0200 110.5700 1.1123

IC CD	CE	NZ	HZ1	1.5350 110.4600 179.9200 110.0200 1.0404

IC HZ1 CE	*NZ HZ2	1.0404 110.0200 120.2700 109.5000 1.0402

IC HZ1 CE	*NZ HZ3	1.0404 110.0200 -120.1300 109.4000 1.0401

RESI MET	0.00

GROUP

ATOM N	NH1	-0.47 ! |

ATOM HN	H	0.31 ! HN-N

ATOM CA	CT1	0.07 ! |	HB1 HG1	HE1

ATOM HA	HB	0.09 ! |	|	|	|

GROUP	! HA-CA--CB--CG--SD--CE--HE3

ATOM CB	CT2	-0.18 ! |	|	|	|

ATOM HB1 HA	0.09 ! |	HB2 HG2	HE2

ATOM HB2 HA	0.09 ! O=C

GROUP	! |

ATOM CG	CT2	-0.14

ATOM HG1 HA	0.09

ATOM HG2 HA	0.09

ATOM SD	S	-0.09

ATOM CE	CT3	-0.22

ATOM HE1 HA	0.09

ATOM HE2 HA	0.09

ATOM HE3 HA	0.09

GROUP

ATOM C	C	0.51

ATOM O	O	-0.51

BOND CB CA	CG CB	SD CG	CE SD

BOND N HN	N CA	C CA	C +N

BOND CA HA	CB HB1 CB HB2 CG HG1 CG HG2

BOND CE HE1 CE HE2 CE HE3

DOUBLE	O C

IMPR N -C CA HN C CA +N O

CMAP -C N CA C	N CA C +N

DONOR HN N

ACCEPTOR O C

IC -C	CA	*N	HN	1.3478 124.2100 180.0000 114.3900 0.9978

IC -C	N	CA	C	1.3478 124.2100 180.0000 106.3100 1.5195

IC N	CA	C	+N	1.4510 106.3100 180.0000 117.7400 1.3471

IC +N	CA	*C	O	1.3471 117.7400 180.0000 120.6400 1.2288

IC CA	C	+N	+CA	1.5195 117.7400 180.0000 124.5200 1.4471

IC N	C	*CA CB	1.4510 106.3100 121.6200 111.8800 1.5546

IC N	C	*CA HA	1.4510 106.3100 -116.9800 107.5700 1.0832

IC N	CA	CB	CG	1.4510 111.2500 180.0000 115.9200 1.5460

IC CG	CA	*CB HB1	1.5460 115.9200 120.5600 106.9000 1.1153

IC CG	CA	*CB HB2	1.5460 115.9200 -124.8000 109.3800 1.1129

IC CA	CB	CG	SD	1.5546 115.9200 180.0000 110.2800 1.8219

IC SD	CB	*CG HG1	1.8219 110.2800 120.5000 110.3400 1.1106

IC SD	CB	*CG HG2	1.8219 110.2800 -121.1600 109.6400 1.1119

IC CB	CG	SD	CE	1.5460 110.2800 180.0000 98.9400 1.8206

IC CG	SD	CE	HE1	1.8219 98.9400 -179.4200 110.9100 1.1111

IC HE1 SD	*CE HE2	1.1111 110.9100 119.9500 111.0300 1.1115

IC HE1 SD	*CE HE3	1.1111 110.9100 -119.9500 111.0900 1.1112

RESI PHE	0.00

GROUP

ATOM N	NH1	-0.47 ! |	HD1 HE1

ATOM HN	H	0.31 ! HN-N	|	|

ATOM CA	CT1	0.07 ! |	HB1 CD1--CE1

ATOM HA	HB	0.09 ! |	|	//	\\

GROUP	! HA-CA--CB--CG	CZ--HZ

ATOM CB	CT2	-0.18 ! |	|	\ __ /

ATOM HB1 HA	0.09 ! |	HB2 CD2--CE2

ATOM HB2 HA	0.09 ! O=C	|	|

GROUP	! |	HD2 HE2

ATOM CG	CA	0.00

GROUP

ATOM CD1 CA	-0.115

ATOM HD1 HP	0.115

GROUP

ATOM CE1 CA	-0.115

ATOM HE1 HP	0.115

GROUP

ATOM CZ	CA	-0.115

ATOM HZ	HP	0.115

GROUP

ATOM CD2 CA	-0.115

ATOM HD2 HP	0.115

GROUP

ATOM CE2 CA	-0.115

ATOM HE2 HP	0.115

GROUP

ATOM C	C	0.51

ATOM O	O	-0.51

BOND CB CA	CG CB	CD2 CG	CE1 CD1

BOND CZ CE2 N	HN

BOND N	CA	C	CA	C	+N	CA HA

BOND CB HB1 CB HB2 CD1 HD1 CD2 HD2 CE1 HE1

DOUBLE	O C	CD1 CG CZ CE1	CE2 CD2

BOND CE2 HE2 CZ HZ

IMPR N -C CA HN C CA +N O

CMAP -C N CA C	N CA C +N

DONOR HN N

ACCEPTOR O C

IC -C	CA	*N	HN	1.3476 123.8900 180.0000 114.4700 0.9987

IC -C	N	CA	C	1.3476 123.8900 180.0000 106.3800 1.5229

IC N	CA	C	+N	1.4504 106.3800 180.0000 117.6500 1.3483

IC +N	CA	*C	O	1.3483 117.6500 180.0000 120.4900 1.2287

IC CA	C	+N	+CA	1.5229 117.6500 180.0000 124.1000 1.4523

IC N	C	*CA CB	1.4504 106.3800 122.4900 112.4500 1.5594

IC N	C	*CA HA	1.4504 106.3800 -115.6300 107.0500 1.0832

IC N	CA	CB	CG	1.4504 111.6300 180.0000 112.7600 1.5109

IC CG	CA	*CB HB1	1.5109 112.7600 118.2700 109.1000 1.1119

IC CG	CA	*CB HB2	1.5109 112.7600 -123.8300 111.1100 1.1113

IC CA	CB	CG	CD1	1.5594 112.7600	90.0000 120.3200 1.4059

IC CD1 CB	*CG CD2	1.4059 120.3200 -177.9600 120.7600 1.4062

IC CB	CG	CD1 CE1	1.5109 120.3200 -177.3700 120.6300 1.4006

IC CE1 CG	*CD1 HD1	1.4006 120.6300 179.7000 119.6500 1.0814

IC CB	CG	CD2 CE2	1.5109 120.7600 177.2000 120.6200 1.4002

IC CE2 CG	*CD2 HD2	1.4002 120.6200 -178.6900 119.9900 1.0811

IC CG	CD1 CE1 CZ	1.4059 120.6300	-0.1200 119.9300 1.4004

IC CZ	CD1 *CE1 HE1	1.4004 119.9300 -179.6900 120.0100 1.0808

IC CZ	CD2 *CE2 HE2	1.4000 119.9600 -179.9300 119.8700 1.0811

IC CE1 CE2 *CZ HZ	1.4004 119.9800 179.5100 119.9700 1.0807

RESI PRO	0.00

GROUP	! HD1 HD2

ATOM N	N	-0.29 ! |	\ /

ATOM CD	CP3	0.00 ! N---CD	HG1 ATOM CA	CP1	0.02

ATOM HD1 HA	0.09 ! |	\ /

ATOM HD2 HA	0.09 ! |	CG

ATOM CA	CP1	0.02 ! |	/ \

ATOM HA	HB	0.09 ! HA-CA--CB	HG2

GROUP	! |	/ \

ATOM CB	CP2	-0.18 ! | HB1 HB2

ATOM HB1 HA	0.09 ! O=C

ATOM HB2 HA	0.09 ! |

GROUP

ATOM CG	CP2	-0.18

ATOM HG1 HA	0.09

ATOM HG2 HA	0.09

GROUP

ATOM C	C	0.51

ATOM O	O	-0.51

BOND C CA C	+N

BOND N CA CA CB CB CG CG CD N	CD

BOND HA CA HG1 CG HG2 CG HD1 CD HD2 CD HB1 CB HB2 CB

DOUBLE	O C

IMPR N -C CA CD

IMPR C CA +N O

CMAP -C N CA C	N CA C +N

ACCEPTOR O C

IC -C	CA	*N	CD	1.3366 122.9400 178.5100 112.7500 1.4624

IC -C	N	CA	C	1.3366 122.9400 -76.1200 110.8600 1.5399

IC N	CA	C	+N	1.4585 110.8600 180.0000 114.7500 1.3569

IC +N	CA	*C	O	1.3569 114.7500 177.1500 120.4600 1.2316

IC CA	C	+N	+CA	1.5399 116.1200 180.0000 124.8900 1.4517

IC N	C	*CA CB	1.4585 110.8600 113.7400 111.7400 1.5399

IC N	C	*CA HA	1.4585 110.8600 -122.4000 109.0900 1.0837

IC N	CA	CB	CG	1.4585 102.5600	31.6100 104.3900 1.5322

IC CA	CB	CG	CD	1.5399 104.3900 -34.5900 103.2100 1.5317

IC N	CA	CB	HB1	1.4585 102.5600 -84.9400 109.0200 1.1131

IC N	CA	CB	HB2	1.4585 102.5600 153.9300 112.7400 1.1088

IC CA	CB	CG	HG1	1.5399 104.3900 -156.7200 112.9500 1.1077

IC CA	CB	CG	HG2	1.5399 104.3900	81.2600 109.2200 1.1143

IC CB	CG	CD	HD1	1.5322 103.2100 -93.5500 110.0300 1.1137

IC CB	CG	CD	HD2	1.5322 103.2100 144.5200 110.0000 1.1144

PATCHING FIRS PROP

RESI SER	0.00

GROUP

ATOM N	NH1	-0.47 ! |

ATOM HN	H	0.31 ! HN-N

ATOM CA	CT1	0.07 ! |	HB1

ATOM HA	HB	0.09 ! |	|

GROUP	! HA-CA--CB--OG

ATOM CB	CT2	0.05 ! |	|	\

ATOM HB1 HA	0.09 ! |	HB2	HG1

ATOM HB2 HA	0.09 ! O=C

ATOM OG	OH1	-0.66 ! |

ATOM HG1 H	0.43

GROUP

ATOM C	C	0.51

ATOM O	O	-0.51

BOND CB CA	OG CB N HN N CA

BOND C CA C +N CA HA CB HB1

BOND CB HB2 OG HG1

DOUBLE	O C

IMPR N -C CA HN C CA +N O

CMAP -C N CA C	N CA C +N

DONOR HN N

DONOR HG1 OG

ACCEPTOR OG

ACCEPTOR O C

IC -C	CA	*N	HN	1.3474 124.3700 180.0000 114.1800 0.9999

IC -C	N	CA	C	1.3474 124.3700 180.0000 105.8100 1.5166

IC N	CA	C	+N	1.4579 105.8100 180.0000 117.7200 1.3448

IC +N	CA	*C	O	1.3448 117.7200 180.0000 120.2500 1.2290

IC CA	C	+N	+CA	1.5166 117.7200 180.0000 124.6300 1.4529

IC N	C	*CA CB	1.4579 105.8100 124.7500 111.4000 1.5585

IC N	C	*CA HA	1.4579 105.8100 -115.5600 107.3000 1.0821

IC N	CA	CB	OG	1.4579 114.2800 180.0000 112.4500 1.4341

IC OG	CA	*CB HB1	1.4341 112.4500 119.3200 108.1000 1.1140

IC OG	CA	*CB HB2	1.4341 112.4500 -123.8600 110.3800 1.1136

IC CA	CB	OG	HG1	1.5585 112.4500 165.9600 107.0800 0.9655

RESI THR	0.00

GROUP

ATOM N	NH1	-0.47 ! |

ATOM HN	H	0.31 ! HN-N

ATOM CA	CT1	0.07 ! |	OG1--HG1

ATOM HA	HB	0.09 ! |	/

GROUP	! HA-CA--CB-HB

ATOM CB	CT1	0.14 ! |	\

ATOM HB	HA	0.09 ! |	CG2--HG21

ATOM OG1 OH1	-0.66 ! O=C	/ \

ATOM HG1 H	0.43 ! | HG21 HG22

GROUP

ATOM CG2 CT3	-0.27

ATOM HG21 HA	0.09

ATOM HG22 HA	0.09

ATOM HG23 HA	0.09

GROUP

ATOM C	C	0.51

ATOM O	O	-0.51

BOND CB CA OG1 CB	CG2 CB	N	HN

BOND N CA	C	CA	C	+N	CA HA

BOND CB HB OG1 HG1 CG2 HG21 CG2 HG22 CG2 HG23

DOUBLE O	C

IMPR N -C CA HN C CA +N O

CMAP -C N CA C	N CA C +N

DONOR HN N

DONOR HG1 OG1

ACCEPTOR OG1

ACCEPTOR O C

IC -C	CA	*N	HN	1.3471 124.1200 180.0000 114.2600 0.9995

IC -C	N	CA	C	1.3471 124.1200 180.0000 106.0900 1.5162

IC N	CA	C	+N	1.4607 106.0900 180.0000 117.6900 1.3449

IC +N	CA	*C	O	1.3449 117.6900 180.0000 120.3000 1.2294

IC CA	C	+N	+CA	1.5162 117.6900 180.0000 124.6600 1.4525

IC N	C	*CA CB	1.4607 106.0900 126.4600 112.7400 1.5693

IC N	C	*CA HA	1.4607 106.0900 -114.9200 106.5300 1.0817

IC N	CA	CB	OG1	1.4607 114.8100 180.0000 112.1600 1.4252

IC OG1 CA	*CB HB	1.4252 112.1600 116.3900 106.1100 1.1174

IC OG1 CA	*CB CG2	1.4252 112.1600 -124.1300 115.9100 1.5324

IC CA	CB	OG1 HG1	1.5693 112.1600 -179.2800 105.4500 0.9633

IC CA	CB	CG2 HG21 1.5693 115.9100 -173.6500 110.8500 1.1104

IC HG21 CB	*CG2 HG22 1.1104 110.8500 119.5100 110.4100 1.1109

IC HG21 CB	*CG2 HG23 1.1104 110.8500 -120.3900 111.1100 1.1113

RESI TRP	0.00

GROUP

ATOM N	NH1	-0.47 ! |	HE3

ATOM HN	H	0.31 ! HN-N	|

ATOM CA	CT1	0.07 ! |	HB1	CE3

ATOM HA	HB	0.09 ! |	|	/ \\

GROUP	! HA-CA--CB---CG-----CD2	CZ3-HZ3

ATOM CB	CT2	-0.18 ! |	|	||	||	|

ATOM HB1 HA	0.09 ! |	HB2 CD1	CE2	CH2-HH2

ATOM HB2 HA	0.09 ! O=C	/	\	/ \ //

GROUP	! |	HD1	NE1	CZ2

ATOM CG	CY	-0.03 ! |	|

ATOM CD1 CA	0.035 ! HE1	HZ2

ATOM HD1 HP	0.115

ATOM NE1 NY	-0.61

ATOM HE1 H	0.38

ATOM CE2 CPT	0.13

ATOM CD2 CPT	-0.02

GROUP

ATOM CE3 CA	-0.115

ATOM HE3 HP	0.115

GROUP

ATOM CZ3 CA	-0.115

ATOM HZ3 HP	0.115

GROUP

ATOM CZ2 CA	-0.115

ATOM HZ2 HP	0.115

GROUP

ATOM CH2 CA	-0.115

ATOM HH2 HP	0.115

GROUP

ATOM C	C	0.51

ATOM O	O	-0.51

BOND CB CA	CG CB	CD2 CG	NE1 CD1

BOND CZ2 CE2

BOND N	HN	N	CA	C	CA	C	+N

BOND CZ3 CH2 CD2 CE3 NE1 CE2 CA HA	CB HB1

BOND CB HB2 CD1 HD1 NE1 HE1 CE3 HE3 CZ2 HZ2

BOND CZ3 HZ3 CH2 HH2

DOUBLE O	C	CD1 CG	CE2 CD2 CZ3 CE3 CH2 CZ2

IMPR N -C CA HN C CA +N O

CMAP -C N CA C	N CA C +N

DONOR HN N

DONOR HE1 NE1

ACCEPTOR O C

IC -C	CA	*N	HN	1.3482 123.5100 180.0000 115.0200 0.9972

IC -C	N	CA	C	1.3482 123.5100 180.0000 107.6900 1.5202

IC N	CA	C	+N	1.4507 107.6900 180.0000 117.5700 1.3505

IC +N	CA	*C	O	1.3505 117.5700 180.0000 121.0800 1.2304

IC CA	C	+N	+CA	1.5202 117.5700 180.0000 124.8800 1.4526

IC N	C	*CA CB	1.4507 107.6900 122.6800 111.2300 1.5560

IC N	C	*CA HA	1.4507 107.6900 -117.0200 106.9200 1.0835

IC N	CA	CB	CG	1.4507 111.6800 180.0000 115.1400 1.5233

IC CG	CA	*CB HB1	1.5233 115.1400 119.1700 107.8400 1.1127

IC CG	CA	*CB HB2	1.5233 115.1400 -124.7300 109.8700 1.1118

IC CA	CB	CG	CD2	1.5560 115.1400	90.0000 123.9500 1.4407

IC CD2 CB	*CG CD1	1.4407 123.9500 -172.8100 129.1800 1.3679

IC CD1 CG	CD2 CE2	1.3679 106.5700	-0.0800 106.6500 1.4126

IC CG	CD2 CE2 NE1	1.4407 106.6500	0.1400 107.8700 1.3746

IC CE2 CG	*CD2 CE3	1.4126 106.6500 179.2100 132.5400 1.4011

IC CE2 CD2 CE3 CZ3	1.4126 120.8000	-0.2000 118.1600 1.4017

IC CD2 CE3 CZ3 CH2	1.4011 118.1600	0.1000 120.9700 1.4019

IC CE3 CZ3 CH2 CZ2	1.4017 120.9700	0.0100 120.8700 1.4030

IC CZ3 CD2 *CE3 HE3	1.4017 118.1600 -179.6200 121.8400 1.0815

IC CH2 CE3 *CZ3 HZ3	1.4019 120.9700 -179.8200 119.4500 1.0811

IC CZ2 CZ3 *CH2 HH2	1.4030 120.8700 -179.9200 119.5700 1.0811

IC CE2 CH2 *CZ2 HZ2	1.3939 118.4200 179.8700 120.0800 1.0790

IC CD1 CE2 *NE1 HE1	1.3752 108.8100 177.7800 124.6800 0.9767

IC CG	NE1 *CD1 HD1	1.3679 110.1000 178.1000 125.4300 1.0820

RESI TYR	0.00

GROUP

ATOM N	NH1	-0.47 ! |	HD1 HE1

ATOM HN	H	0.31 ! HN-N	|	|

ATOM CA	CT1	0.07 ! |	HB1 CD1--CE1

ATOM HA	HB	0.09 ! |	|	//	\\

GROUP	! HA-CA--CB--CG	CZ--OH

ATOM CB	CT2	-0.18 ! |	|	\ __ /	\

ATOM HB1 HA	0.09 ! |	HB2 CD2--CE2	HH

ATOM HB2 HA	0.09 ! O=C	|	|

GROUP	! |	HD2 HE2

ATOM CG	CA	0.00

GROUP

ATOM CD1 CA	-0.115

ATOM HD1 HP	0.115

GROUP

ATOM CE1 CA	-0.115

ATOM HE1 HP	0.115

GROUP

ATOM CZ	CA	0.11

ATOM OH	OH1	-0.54

ATOM HH	H	0.43

GROUP

ATOM CD2 CA	-0.115

ATOM HD2 HP	0.115

GROUP

ATOM CE2 CA	-0.115

ATOM HE2 HP	0.115

GROUP

ATOM C	C	0.51

ATOM O	O	-0.51

BOND CB CA	CG CB	CD2 CG	CE1 CD1

BOND CZ CE2 OH CZ

BOND N	HN	N	CA	C	CA	C	+N

BOND CA HA	CB HB1 CB HB2 CD1 HD1 CD2 HD2

BOND CE1 HE1 CE2 HE2 OH HH

DOUBLE	O	C	CD1 CG CE1 CZ CE2 CD2

IMPR N -C CA HN C CA +N O

CMAP -C N CA C	N CA C +N

DONOR HN N

DONOR HH OH

ACCEPTOR OH

ACCEPTOR O C

IC -C	CA	*N	HN	1.3476 123.8100 180.0000 114.5400 0.9986

IC -C	N	CA	C	1.3476 123.8100 180.0000 106.5200 1.5232

IC N	CA	C	+N	1.4501 106.5200 180.0000 117.3300 1.3484

IC +N	CA	*C	O	1.3484 117.3300 180.0000 120.6700 1.2287

IC CA	C	+N	+CA	1.5232 117.3300 180.0000 124.3100 1.4513

IC N	C	*CA CB	1.4501 106.5200 122.2700 112.3400 1.5606

IC N	C	*CA HA	1.4501 106.5200 -116.0400 107.1500 1.0833

IC N	CA	CB	CG	1.4501 111.4300 180.0000 112.9400 1.5113

IC CG	CA	*CB HB1	1.5113 112.9400 118.8900 109.1200 1.1119

IC CG	CA	*CB HB2	1.5113 112.9400 -123.3600 110.7000 1.1115

IC CA	CB	CG	CD1	1.5606 112.9400	90.0000 120.4900 1.4064

IC CD1 CB	*CG CD2	1.4064 120.4900 -176.4600 120.4600 1.4068

IC CB	CG	CD1 CE1	1.5113 120.4900 -175.4900 120.4000 1.4026

IC CE1 CG	*CD1 HD1	1.4026 120.4000 178.9400 119.8000 1.0814

IC CB	CG	CD2 CE2	1.5113 120.4600 175.3200 120.5600 1.4022

IC CE2 CG	*CD2 HD2	1.4022 120.5600 -177.5700 119.9800 1.0813

IC CG	CD1 CE1 CZ	1.4064 120.4000	-0.1900 120.0900 1.3978

IC CZ	CD1 *CE1 HE1	1.3978 120.0900 179.6400 120.5800 1.0799

IC CZ	CD2 *CE2 HE2	1.3979 119.9200 -178.6900 119.7600 1.0798

IC CE1 CE2 *CZ OH	1.3978 120.0500 -178.9800 120.2500 1.4063

IC CE1 CZ	OH	HH	1.3978 119.6800 175.4500 107.4700 0.9594

RESI VAL	0.00

GROUP

ATOM N	NH1	-0.47 ! |	HG11 HG12

ATOM HN	H	0.31 ! HN-N	| /

ATOM CA	CT1	0.07 ! |	CG1--HG13

ATOM HA	HB	0.09 ! |	/

GROUP	! HA-CA--CB-HB

ATOM CB	CT1	-0.09 ! |	\

ATOM HB	HA	0.09 ! |	CG2--HG21

GROUP	! O=C	/ \

ATOM CG1 CT3	-0.27 ! | HG21 HG22

ATOM HG11 HA	0.09

ATOM HG12 HA	0.09

ATOM HG13 HA	0.09

GROUP

ATOM CG2 CT3	-0.27

ATOM HG21 HA	0.09

ATOM HG22 HA	0.09

ATOM HG23 HA	0.09

GROUP

ATOM C	C	0.51

ATOM O	O	-0.51

BOND CB CA	CG1 CB	CG2 CB	N	HN

BOND N	CA	C	CA	C	+N	CA HA

BOND CB HB	CG1 HG11 CG1 HG12 CG1 HG13 CG2 HG21

BOND CG2 HG22 CG2 HG23

DOUBLE	O	C

IMPR N -C CA HN C CA +N O

CMAP -C N CA C	N CA C +N

DONOR HN N

ACCEPTOR O C

IC -C	CA	*N	HN	1.3482 124.5700 180.0000 114.4100 0.9966

IC -C	N	CA	C	1.3482 124.5700 180.0000 105.5400 1.5180

IC N	CA	C	+N	1.4570 105.5400 180.0000 117.8300 1.3471

IC +N	CA	*C	O	1.3471 117.8300 180.0000 120.7000 1.2297

IC CA	C	+N	+CA	1.5180 117.8300 180.0000 124.0800 1.4471

IC N	C	*CA CB	1.4570 105.5400 122.9500 111.2300 1.5660

IC N	C	*CA HA	1.4570 105.5400 -117.2400 107.4600 1.0828

IC N	CA	CB	CG1	1.4570 113.0500 180.0000 113.9700 1.5441

IC CG1 CA	*CB CG2	1.5441 113.9700 123.9900 112.1700 1.5414

IC CG1 CA	*CB HB	1.5441 113.9700 -119.1700 107.5700 1.1178

IC CA	CB	CG1 HG11 1.5660 113.9700 177.8300 110.3000 1.1114

IC HG11 CB	*CG1 HG12 1.1114 110.3000 119.2500 111.6700 1.1097

IC HG11 CB	*CG1 HG13 1.1114 110.3000 -119.4900 110.7000 1.1110

IC CA	CB	CG2 HG21 1.5660 112.1700 -177.7800 110.7100 1.1108

IC HG21 CB	*CG2 HG22 1.1108 110.7100 120.0800 110.5600 1.1115

IC HG21 CB	*CG2 HG23 1.1108 110.7100 -119.5500 111.2300 1.1098

RESI ALAD	0.00 ! Alanine dipeptide, with CMAP term

GROUP

ATOM CL	CT3	-0.27

ATOM HL1 HA	0.09

ATOM HL2 HA	0.09

ATOM HL3 HA	0.09

GROUP

ATOM CLP C	0.51

ATOM OL	O	-0.51

GROUP

ATOM NL	NH1	-0.47

ATOM HL	H	0.31

ATOM CA	CT1	0.07

ATOM HA	HB	0.09

GROUP

ATOM CB	CT3	-0.27 ! HL1	OL	OR	HR1

ATOM HB1 HA	0.09 ! \	||	HL HA ||	HR	/

ATOM HB2 HA	0.09 ! \	||	|	|	||	|	/

ATOM HB3 HA	0.09 ! HL2---CL--CLP--NL--CA--CRP--NR---CR---HR2

GROUP	! /	|	\

ATOM CRP C	0.51 ! /	HB1--CB--HB3	\

ATOM OR	O	-0.51 ! HL3	|	HR3

GROUP	! HB2

ATOM NR	NH1	-0.47

ATOM HR	H	0.31

ATOM CR	CT3	-0.11

ATOM HR1 HA	0.09

ATOM HR2 HA	0.09

ATOM HR3 HA	0.09

BOND CL CLP	CLP NL	NL CA

BOND CA CRP	CRP NR	NR CR

DOUBLE CLP OL	CRP OR

BOND NL HL	NR HR

BOND CA HA	CA CB

BOND CL HL1	CL HL2	CL HL3

BOND CB HB1	CB HB2	CB HB3

BOND CR HR1	CR HR2	CR HR3

IMPR CLP CL NL OL	NL CLP CA HL

IMPR CRP CA NR OR	NR CRP CR HR

CMAP CLP NL CA CRP NL CA CRP NR

ic clp nl ca crp 0.0 0.0 180.0 0.0 0.0 ! Phi

ic ca clp *nl hl	0.0 0.0 180.0 0.0 0.0

ic hl nl ca crp 0.0 0.0	0.0 0.0 0.0

ic nl ca crp nr	0.0 0.0 180.0 0.0 0.0 ! Psi

ic ca nr *crp or 0.0 0.0 180.0 0.0 0.0

ic nl ca crp or	0.0 0.0	0.0 0.0 0.0

ic cl clp nl ca	0.0 0.0 180.0 0.0 0.0 ! Omega Left

ic nl cl *clp ol 0.0 0.0 180.0 0.0 0.0

ic ol clp nl ca	0.0 0.0	0.0 0.0 0.0

ic ca crp nr cr	0.0 0.0 180.0 0.0 0.0 ! Omega Right

ic crp cr *nr hr	0.0 0.0 180.0 0.0 0.0

ic ca crp nr hr	0.0 0.0 180.0 0.0 0.0

ic nl crp *ca ha	0.0 0.0 240.0 0.0 0.0

ic nl crp *ca cb	0.0 0.0 120.0 0.0 0.0

ic hl1 cl clp nl	0.0 0.0 180.0 0.0 0.0

ic hl2 cl clp nl	0.0 0.0	60.0 0.0 0.0

ic hl3 cl clp ol	0.0 0.0 120.0 0.0 0.0

ic ha ca cb hb1 0.0 0.0 180.0 0.0 0.0

ic nl ca cb hb2 0.0 0.0 180.0 0.0 0.0

ic crp ca cb hb3 0.0 0.0 180.0 0.0 0.0

ic crp nr cr hr1 0.0 0.0 180.0 0.0 0.0

ic crp nr cr hr2 0.0 0.0	60.0 0.0 0.0

ic hr nr cr hr3 0.0 0.0 120.0 0.0 0.0

ic ca clp *nl hl	0.0 0.0 180.0 0.0 0.0

ic ca nr *crp or 0.0 0.0 180.0 0.0 0.0

ic hb1 hb2 *cb hb3 0.0 0.0 120.0 0.0 0.0

ic hl1 hl2 *cl hl3 0.0 0.0 240.0 0.0 0.0

ic hr1 hr2 *cr hr3 0.0 0.0 240.0 0.0 0.0

ic ha ca nl hl	0.0 0.0 240.0 0.0 0.0

patch first none last none

RESI TIP3	0.000 ! tip3p water model, generate using noangle nodihedral

GROUP

ATOM OH2 OT	-0.834

ATOM H1	HT	0.417

ATOM H2	HT	0.417

BOND OH2 H1 OH2 H2 H1 H2	! the last bond is needed for shake

ANGLE H1 OH2 H2	! required

ACCEPTOR OH2

PATCHING FIRS NONE LAST NONE

RESI TP3M	0.000 ! "mmff" water model, as an analog of tip3p

GROUP

ATOM OH2 OT	-0.834 ! these charges are replaced by the mmff setup

ATOM H1	HT	0.417 ! these charges are replaced by the mmff setup

ATOM H2	HT	0.417 ! these charges are replaced by the mmff setup

BOND OH2 H1 OH2 H2	! omits the H1-H2 bond, which is needed for shake with tip3p

ANGLE H1 OH2 H2	! required

ACCEPTOR OH2

PATCHING FIRS NONE LAST NONE

! Ion parameters from Benoit Roux and Coworkers

! As of 8/98 no NBFIX terms required

!

RESI SOD	1.00 ! Sodium Ion

GROUP

ATOM SOD SOD 1.00

PATCHING FIRST NONE LAST NONE

RESI MG	2.00 ! Magnesium Ion

GROUP

ATOM MG	MG	2.00

PATCHING FIRST NONE LAST NONE

RESI POT	1.00 ! Potassium Ion

GROUP

ATOM POT	POT 1.00

PATCHING FIRST NONE LAST NONE

RESI CES	1.00 ! Cesium Ion

GROUP

ATOM CES CES 1.00

PATCHING FIRST NONE LAST NONE

RESI CAL	2.00 ! Calcium Ion

GROUP

ATOM CAL CAL 2.00

PATCHING FIRST NONE LAST NONE

RESI CLA	-1.00 ! Chloride Ion

GROUP

ATOM CLA CLA -1.00

PATCHING FIRST NONE LAST NONE

RESI ZN2	2.00 ! Zinc ion, Roland Stote

GROUP

ATOM ZN	ZN	2.00

PATCHING FIRST NONE LAST NONE

PRES NTER	1.00 ! standard N-terminus

GROUP	! use in generate statement

ATOM N	NH3	-0.30 !

ATOM HT1 HC	0.33 ! HT1

ATOM HT2 HC	0.33 ! (+)/

ATOM HT3 HC	0.33 ! --CA--N--HT2

ATOM CA	CT1	0.21 ! |	\

ATOM HA	HB	0.10 ! HA	HT3

DELETE ATOM HN

BOND HT1 N HT2 N HT3 N

DONOR HT1 N

DONOR HT2 N

DONOR HT3 N

IC HT1 N	CA	C	0.0000 0.0000 180.0000 0.0000 0.0000

IC HT2 CA	*N	HT1	0.0000 0.0000 120.0000 0.0000 0.0000

IC HT3 CA	*N	HT2	0.0000 0.0000 120.0000 0.0000 0.0000

PRES GLYP	1.00 ! Glycine N-terminus

GROUP	! use in generate statement

ATOM N	NH3	-0.30 !

ATOM HT1 HC	0.33 ! HA1	HT1

ATOM HT2 HC	0.33 ! | (+)/

ATOM HT3 HC	0.33 ! --CA--N--HT2

ATOM CA	CT2	0.13 ! |	\

ATOM HA1 HB	0.09 ! HA2	HT3

ATOM HA2 HB	0.09 !

DELETE ATOM HN

BOND HT1 N HT2 N HT3 N

DONOR HT1 N

DONOR HT2 N

DONOR HT3 N

IC HT1 N	CA	C	0.0000 0.0000 180.0000 0.0000 0.0000

IC HT2 CA	*N	HT1	0.0000 0.0000 120.0000 0.0000 0.0000

IC HT3 CA	*N	HT2	0.0000 0.0000 120.0000 0.0000 0.0000

PRES PROP	1.00 ! Proline N-Terminal

GROUP	! use in generate statement

ATOM N	NP	-0.07 ! HA

ATOM HN1 HC	0.24 ! |

ATOM HN2 HC	0.24 ! -CA	HN1

ATOM CD	CP3	0.16 ! / \ /

ATOM HD1 HA	0.09 ! N(+)

ATOM HD2 HA	0.09 ! / \

ATOM CA	CP1	0.16 ! -CD	HN2

ATOM HA	HB	0.09 ! | \

BOND HN1 N HN2 N	! HD1 HD2

DONOR HN1 N

DONOR HN2 N

IC HN1 CA	*N	CD	0.0000 0.0000 120.0000 0.0000 0.0000

IC HN2 CA	*N	HN1	0.0000 0.0000 120.0000 0.0000 0.0000

PRES ACE	0.00 ! acetylated N-terminus

! do NOT use to create dipeptides, see ACED

GROUP	! use in generate statement

ATOM CAY CT3	-0.27 !

ATOM HY1 HA	0.09 ! HY1 HY2 HY3

ATOM HY2 HA	0.09 ! \ | /

ATOM HY3 HA	0.09 ! CAY

GROUP	! |

ATOM CY	C	0.51 ! CY=OY

ATOM OY	O	-0.51 ! |

!

BOND CY CAY CY N CAY HY1 CAY HY2 CAY HY3

DOUBLE OY CY

IMPR CY CAY N OY

IMPR N CY CA HN

CMAP CY N CA C	N CA C +N

ACCEPTOR OY CY

IC CY	N	CA	C	0.0000 0.0000 -60.0000 0.0000 0.0000

IC CY	CA	*N	HN	0.0000 0.0000 180.0000 0.0000 0.0000

IC CAY CY	N	CA	0.0000 0.0000 180.0000 0.0000 0.0000

IC N	CAY *CY OY	0.0000 0.0000 180.0000 0.0000 0.0000

IC OY	CY	CAY HY1	0.0000 0.0000 180.0000 0.0000 0.0000

IC OY	CY	CAY HY2	0.0000 0.0000	60.0000 0.0000 0.0000

IC OY	CY	CAY HY3	0.0000 0.0000 -60.0000 0.0000 0.0000

PRES ACED	0.00 ! acetylated N-terminus (to create dipeptide)

GROUP	! use in generate statement

ATOM CAY CT3	-0.27 !

ATOM HY1 HA	0.09 ! HY1 HY2 HY3

ATOM HY2 HA	0.09 ! \ | /

ATOM HY3 HA	0.09 ! CAY

GROUP	! |

ATOM CY	C	0.51 ! CY=OY

ATOM OY	O	-0.51 ! |

!

BOND CY CAY CY N CAY HY1 CAY HY2 CAY HY3

DOUBLE OY CY

IMPR CY CAY N OY

IMPR N CY CA HN

CMAP CY N CA C	N CA C NT

ACCEPTOR OY CY

IC CY	N	CA	C	0.0000 0.0000 -60.0000 0.0000 0.0000

IC CY	CA	*N	HN	0.0000 0.0000 180.0000 0.0000 0.0000

IC CAY CY	N	CA	0.0000 0.0000 180.0000 0.0000 0.0000

IC N	CAY *CY OY	0.0000 0.0000 180.0000 0.0000 0.0000

IC OY	CY	CAY HY1	0.0000 0.0000 180.0000 0.0000 0.0000

IC OY	CY	CAY HY2	0.0000 0.0000	60.0000 0.0000 0.0000

IC OY	CY	CAY HY3	0.0000 0.0000 -60.0000 0.0000 0.0000

PRES ACP	0.00 ! acetylated N-terminus

! NOT for proline dipeptide, see ACPD

GROUP	! use in generate statement

ATOM CAY CT3	-0.27 !

ATOM HY1 HA	0.09 ! HY1 HY2 HY3

ATOM HY2 HA	0.09 ! \ | /

ATOM HY3 HA	0.09 ! CAY

GROUP	! |

ATOM CY	C	0.51 ! CY=OY

ATOM OY	O	-0.51 ! |

!

BOND CY CAY CY N CAY HY1 CAY HY2 CAY HY3

DOUBLE OY CY

IMPR CY CAY N OY

IMPR N CY CA CD

CMAP CY N CA C	N CA C +N

ACCEPTOR OY CY

IC CY	N	CA	C	0.0000 0.0000 -60.0000 0.0000 0.0000

IC CY	CA	*N	CD	0.0000 0.0000 180.0000 0.0000 0.0000

IC CAY CY	N	CA	0.0000 0.0000 180.0000 0.0000 0.0000

IC N	CAY *CY OY	0.0000 0.0000 180.0000 0.0000 0.0000

IC OY	CY	CAY HY1	0.0000 0.0000 180.0000 0.0000 0.0000

IC OY	CY	CAY HY2	0.0000 0.0000	60.0000 0.0000 0.0000

IC OY	CY	CAY HY3	0.0000 0.0000 -60.0000 0.0000 0.0000

PRES ACPD	0.00 ! acetylated N-terminus (for proline dipeptide)

GROUP	! use in generate statement

ATOM CAY CT3	-0.27 !

ATOM HY1 HA	0.09 ! HY1 HY2 HY3

ATOM HY2 HA	0.09 ! \ | /

ATOM HY3 HA	0.09 ! CAY

GROUP	! |

ATOM CY	C	0.51 ! CY=OY

ATOM OY	O	-0.51 ! |

!

BOND CY CAY CY N CAY HY1 CAY HY2 CAY HY3

DOUBLE OY CY

IMPR CY CAY N OY

IMPR N CY CA CD

CMAP CY N CA C	N CA C NT

ACCEPTOR OY CY

IC CY	N	CA	C	0.0000 0.0000 -60.0000 0.0000 0.0000

IC CY	CA	*N	CD	0.0000 0.0000 180.0000 0.0000 0.0000

IC CAY CY	N	CA	0.0000 0.0000 180.0000 0.0000 0.0000

IC N	CAY *CY OY	0.0000 0.0000 180.0000 0.0000 0.0000

IC OY	CY	CAY HY1	0.0000 0.0000 180.0000 0.0000 0.0000

IC OY	CY	CAY HY2	0.0000 0.0000	60.0000 0.0000 0.0000

IC OY	CY	CAY HY3	0.0000 0.0000 -60.0000 0.0000 0.0000

PRES NNEU	0.00 ! neutral N-terminus; charges from LSN

GROUP	! use in generate statement

ATOM N	NH2	-0.96 !

ATOM HT1 H	0.34 ! HT1

ATOM HT2 H	0.34 ! /

! --CA--N--HT2

ATOM CA	CT1	0.19 ! |	! change to CT2 for neutral N terminal glycine

ATOM HA	HB	0.09 ! HA	! change to HA1 and HB2 and add HA2 atom for N terminal glycine

DELETE ATOM HN

BOND HT1 N HT2 N

DONOR HT1 N

DONOR HT2 N

IC HT1 N	CA	C	0.0000 0.0000 180.0000 0.0000 0.0000

IC HT2 CA	*N	HT1	0.0000 0.0000 120.0000 0.0000 0.0000

PRES CTER	-1.00 ! standard C-terminus

GROUP	! use in generate statement

ATOM C	CC	0.34 ! OT2(-)

ATOM OT1 OC	-0.67 ! /

ATOM OT2 OC	-0.67 ! -C

DELETE ATOM O	! \\

BOND C OT2	! OT1

DOUBLE C OT1

IMPR C CA OT2 OT1

ACCEPTOR OT1 C

ACCEPTOR OT2 C

IC N	CA	C	OT2	0.0000 0.0000 180.0000 0.0000 0.0000

IC OT2 CA	*C	OT1	0.0000 0.0000 180.0000 0.0000 0.0000

PRES CT1	0.00 ! methylated C-terminus from methyl acetate

GROUP	! use in generate statement

ATOM N	NH1	-0.47 !

ATOM HN	H	0.31 ! OT1

ATOM CA	CT1	0.17 ! |	//

ATOM HA	HB	0.09 ! -N--CA--C	HT1

ATOM C	CD	0.63 ! | |	\	/

ATOM OT1 OB	-0.52 ! HN HA	OT2--CT--HT2

ATOM OT2 OS	-0.34 ! \

ATOM CT	CT3	-0.14 ! HT3

ATOM HT1 HA	0.09 !

ATOM HT2 HA	0.09 !

ATOM HT3 HA	0.09 !

DELETE ATOM O

BOND C OT2 OT2 CT

BOND CT HT1 CT HT2 CT HT3

DOUBLE C OT1

IMPR C CA OT2 OT1

ACCEPTOR OT1 C

ACCEPTOR OT2 C

IC N	CA	C	OT2	0.0000 0.0000 180.0000 0.0000 0.0000

IC OT2 CA	*C	OT1	0.0000 0.0000 180.0000 0.0000 0.0000

IC CA	C	OT2 CT	0.0000 0.0000 180.0000 0.0000 0.0000

IC C	OT2 CT	HT1	0.0000 0.0000	0.0000 0.0000 0.0000

IC C	OT2 CT	HT2	0.0000 0.0000 120.0000 0.0000 0.0000

IC C	OT2 CT	HT3	0.0000 0.0000 240.0000 0.0000 0.0000

PRES CT2	0.00 ! amidated C-terminus

GROUP	! use in generate statement

ATOM C	CC	0.55 !

ATOM O	O	-0.55 ! |

GROUP	! O=C

ATOM NT	NH2	-0.62 ! |

ATOM HT1 H	0.32 ! NT

ATOM HT2 H	0.30 ! / \

BOND C NT	! HT1 HT2 (HT1 is cis to O)

BOND NT HT1 NT HT2	!

IMPR C NT CA O C CA NT O

IMPR NT C HT1 HT2 NT C HT2 HT1

DONOR HT1 NT

DONOR HT2 NT

IC N	CA	C	O	0.0000 0.0000 180.0000 0.0000 0.0000

IC NT	CA	*C	O	0.0000 0.0000 180.0000 0.0000 0.0000

IC CA	C	NT	HT1	0.0000 0.0000 180.0000 0.0000 0.0000

IC HT1 C	*NT HT2	0.0000 0.0000 180.0000 0.0000 0.0000

PRES CT3	0.00 ! N-Methylamide C-terminus

GROUP	! use in generate statement

ATOM C	C	0.51 !

ATOM O	O	-0.51 ! |

GROUP	! C=O

ATOM NT	NH1	-0.47 ! |

ATOM HNT H	0.31 ! NT-HNT

ATOM CAT CT3	-0.11 ! |

ATOM HT1 HA	0.09 ! HT1-CAT-HT3

ATOM HT2 HA	0.09 ! |

ATOM HT3 HA	0.09 ! HT2

!

BOND C NT NT HNT NT CAT CAT HT1 CAT HT2 CAT HT3

IMPR NT C CAT HNT C CA NT O

CMAP -C N CA C	N CA C NT

DONOR HNT NT

IC N	CA	C	O	0.0000 0.0000 180.0000 0.0000 0.0000

IC NT	CA	*C	O	0.0000 0.0000 180.0000 0.0000 0.0000

IC C	CAT *NT HNT	0.0000 0.0000 180.0000 0.0000 0.0000

IC CA	C	NT	CAT	0.0000 0.0000 180.0000 0.0000 0.0000

IC C	NT	CAT HT1	0.0000 0.0000	60.0000 0.0000 0.0000

IC C	NT	CAT HT2	0.0000 0.0000 180.0000 0.0000 0.0000

IC C	NT	CAT HT3	0.0000 0.0000 -60.0000 0.0000 0.0000

PRES CNEU	0.00 ! neutral C-terminus; charges from ASPP

GROUP	! use in generate statement; C reduced to balance

ATOM C	CD	0.72 ! OT2-HT2

ATOM OT1 OB	-0.55 ! /

ATOM OT2 OH1	-0.61 ! -C

ATOM HT2 H	0.44 ! \\

DELETE ATOM O	! OT1

BOND C OT2	!

BOND OT2 HT2

DOUBLE C OT1

IMPR C CA OT2 OT1

ACCEPTOR OT1 C

ACCEPTOR OT2 C

IC N	CA	C	OT2	0.0000 0.0000 180.0000 0.0000 0.0000

IC OT2 CA	*C	OT1	0.0000 0.0000 180.0000 0.0000 0.0000

IC CA	C	OT2 HT2	0.0000 0.0000 180.0000 0.0000 0.0000

PRES ASPP	0.00 ! patch for protonated aspartic acid, proton on od2

GROUP	! via acetic acid, use in a patch statementand

! follow with AUTOgenerate ANGLes DIHEdrals command

ATOM CB	CT2	-0.21 !

ATOM HB1 HA	0.09 ! HB1	OD1

ATOM HB2 HA	0.09 ! |	//

ATOM CG	CD	0.75 ! -CB--CG

ATOM OD1 OB	-0.55 ! |	\

ATOM OD2 OH1	-0.61 ! HB2	OD2-HD2

ATOM HD2 H	0.44 !

BOND OD2 HD2

DONOR HD2 OD2

IC HD2 OD2 CG	OD1	0.0000 0.0000 0.0000 0.0000 0.0000

PRES GLUP	0.00 ! patch for protonated glutamic acid, proton on oe2

GROUP	! via acetic acid, use in a patch statement and

! follow with AUTOgenerate ANGLes DIHEdrals command

ATOM CG	CT2	-0.21 !

ATOM HG1 HA	0.09 ! HG1	OE1

ATOM HG2 HA	0.09 ! |	//

ATOM CD	CD	0.75 ! -CG--CD

ATOM OE1 OB	-0.55 ! |	\

ATOM OE2 OH1	-0.61 ! HG2	OE2-HE2

ATOM HE2 H	0.44 !

BOND OE2 HE2

DONOR HE2 OE2

IC HE2 OE2 CD	OE1	0.0000 0.0000 0.0000 0.0000 0.0000

PRES LSN	0.00 ! patch for neutral lysine based on methylamine

! use in a patch statement

! follow with AUTOgenerate ANGLes DIHEdrals command

!delete atom and reassign charges

DELETE ATOM HZ3

GROUP

ATOM CE	CT2	0.13

ATOM HE1 HA	0.075

ATOM HE2 HA	0.075

ATOM NZ	NH2	-0.96

ATOM HZ1 HC	0.34

ATOM HZ2 HC	0.34

PRES LINK	0.00 ! linkage for IMAGES or for joining segments

! 1 refers to previous (N terminal)

! 2 refers to next (C terminal)

! use in a patch statement

! follow with AUTOgenerate ANGLes DIHEdrals command

BOND 1C 2N

!the need for the explicit specification of angles and dihedrals in

!patches linking images has not been tested

!ANGLE 1C 2N 2CA 1CA 1C 2N

!ANGLE 1O 1C 2N	1C 2N 2HN

!DIHE 1C 2N 2CA 2C	1C 2N 2CA 2HA 1C 2N 2CA 2CB

!DIHE 1HA 1CA 1C 2N	1N 1CA 1C 2N	1CB 1CA 1C 2N

!DIHE 1CA 1C 2N 2HN 1CA 1C 2N 2CA

!DIHE 1O 1C 2N 2HN 1O 1C 2N 2CA

IMPR 2N 1C 2CA 2HN 1C 1CA 2N 1O

IC 1N	1CA 1C	2N	0.0000 0.0000 180.0000 0.0000 0.0000

IC 2N	1CA *1C 1O	0.0000 0.0000 180.0000 0.0000 0.0000

IC 1CA 1C	2N	2CA	0.0000 0.0000 180.0000 0.0000 0.0000

IC 1C	2N	2CA 2C	0.0000 0.0000 180.0000 0.0000 0.0000

IC 1C	2CA *2N 2HN	0.0000 0.0000 180.0000 0.0000 0.0000

PRES DISU	-0.36 ! patch for disulfides. Patch must be 1-CYS and 2-CYS.

! use in a patch statement

! follow with AUTOgenerate ANGLes DIHEdrals command

GROUP

ATOM 1CB CT2	-0.10 !

ATOM 1SG SM	-0.08 ! 2SG--2CB--

GROUP	! /

ATOM 2SG SM	-0.08 ! -1CB--1SG

ATOM 2CB CT2	-0.10 !

DELETE ATOM 1HG1

DELETE ATOM 2HG1

BOND 1SG 2SG

IC 1CA 1CB 1SG 2SG	0.0000 0.0000 180.0000 0.0000 0.0000

IC 1CB 1SG 2SG 2CB	0.0000 0.0000	90.0000 0.0000 0.0000

IC 1SG 2SG 2CB 2CA	0.0000 0.0000 180.0000 0.0000 0.0000

PRES HS2	0.00 ! Patch for neutral His, move proton from ND1 to NE2

! use in a patch statement

! follow with AUTOgenerate ANGLes DIHEdrals command

GROUP

ATOM CE1 CPH2	0.25 ! HE1

ATOM HE1 HR1	0.13 ! /

ATOM ND1 NR2	-0.70 ! HB1	ND1--CE1

ATOM CG	CPH1	0.22 ! |	/	|

ATOM CB	CT2	-0.08 ! -CB--CG	|

ATOM HB1 HA	0.09 ! |	\	|

ATOM HB2 HA	0.09 ! HB2	CD2--NE2

GROUP	! |	\

ATOM NE2 NR1	-0.36 ! HD2	HE2

ATOM HE2 H	0.32

ATOM CD2 CPH1	-0.05

ATOM HD2 HR3	0.09

DELETE ATOM HD1

DELETE ACCE NE2

BOND NE2 HE2

IMPR NE2 CD2 CE1 HE2 NE2 CE1 CD2 HE2

DONOR HE2 NE2

ACCEPTOR ND1

IC CE1 CD2 *NE2 HE2	0.0000 0.0000 180.0000 0.0000 0.0000

! patches for cyclic peptides

PRES LIG1	0.00000 ! linkage for cyclic peptide

! 1 refers to the C terminus which is a glycine

! 2 refers to the N terminus

! use in a patch statement, perform initial

! generation using first NONE last NONE

! follow with AUTOgenerate ANGLes DIHEdrals command

BOND 1C 2N

IMPR 2N 1C 2CA 2HN 1C 1CA 2N 1O

IC 1N	1CA 1C	2N	0.0000 0.0000 180.0000 0.0000 0.0000

IC 2N	1CA *1C 1O	0.0000 0.0000 180.0000 0.0000 0.0000

IC 1CA 1C	2N	2CA	0.0000 0.0000 180.0000 0.0000 0.0000

IC 1C	2N	2CA 2C	0.0000 0.0000 180.0000 0.0000 0.0000

IC 1C	2CA *2N 2HN	0.0000 0.0000 180.0000 0.0000 0.0000

PRES LIG2	0.00000 ! linkage for cyclic peptide

! 1 refers to the C terminus

! 2 refers to the N terminus which is a glycine

! use in a patch statement, perform initial

! generation using first NONE last NONE

! follow with AUTOgenerate ANGLes DIHEdrals command

BOND 1C 2N

IMPR 2N 1C 2CA 2HN 1C 1CA 2N 1O

IC 1N	1CA 1C	2N	0.0000 0.0000 180.0000 0.0000 0.0000

IC 2N	1CA *1C 1O	0.0000 0.0000 180.0000 0.0000 0.0000

IC 1CA 1C	2N	2CA	0.0000 0.0000 180.0000 0.0000 0.0000

IC 1C	2N	2CA 2C	0.0000 0.0000 180.0000 0.0000 0.0000

IC 1C	2CA *2N 2HN	0.0000 0.0000 180.0000 0.0000 0.0000

PRES LIG3	0.00000 ! linkage for cyclic peptide

! 1 refers to the C terminus which is a glycine

! 2 refers to the N terminus which is a glycine

! use in a patch statement, perform initial

! generation using first NONE last NONE

! follow with AUTOgenerate ANGLes DIHEdrals command

BOND 1C 2N

IMPR 2N 1C 2CA 2HN 1C 1CA 2N 1O

IC 1N	1CA 1C	2N	0.0000 0.0000 180.0000 0.0000 0.0000

IC 2N	1CA *1C 1O	0.0000 0.0000 180.0000 0.0000 0.0000

IC 1CA 1C	2N	2CA	0.0000 0.0000 180.0000 0.0000 0.0000

IC 1C	2N	2CA 2C	0.0000 0.0000 180.0000 0.0000 0.0000

IC 1C	2CA *2N 2HN	0.0000 0.0000 180.0000 0.0000 0.0000

END
